# Effects of study design parameters on estimates of bee abundance and richness in agroecosystems: a meta-analysis

**DOI:** 10.1093/aesa/saae001

**Published:** 2024-01-19

**Authors:** Hannah K Levenson, Bradley N Metz, David R Tarpy

**Affiliations:** Department of Entomology and Plant Pathology, North Carolina State University, Raleigh, NC, USA; Department of Applied Ecology, North Carolina State University, Raleigh, NC, USA; Department of Applied Ecology, North Carolina State University, Raleigh, NC, USA

**Keywords:** agricultural entomology, sampling and detection, pollination

## Abstract

Pollinators are critical for agricultural production and food security, leading to many ongoing surveys of pollinators (especially bees) in crop and adjacent landscapes. These surveys have become increasingly important to better understand the community of potential pollinators, quantify relative insect abundance, and secure crop ecosystem services. However, as some bee populations are declining, there is a need to align and improve bee survey efforts, so that they can best meet research and conservation goals, particularly in light of the logistical and financial constraints of conducting such studies. Here, we mined the existing literature on bee surveys in or around agricultural lands to better understand how sampling methods can be optimized to maximize estimates of 2 key measures of bee communities (abundance and richness). After reviewing 72 papers spanning 20 yr of publication, we found that study duration, number of sites, sampling time, and sampling method most significantly influenced abundance, while the number of trips per year and collection method significantly influenced richness. Our analysis helps to derive thresholds, priorities, and recommendations that can be applied to future studies describing bee communities in agroecosystems.

## Introduction

There are more than 20,000 different bee species in the world (Michener 2007) and that number is growing as more species continue to be discovered (Orr et al. 2016, Engel 2022). New bee-species checklists are being written for states across the United States and the world with many reporting and documenting dozens of bee species in their vicinities for the first time. Indeed, 16 newly reported species were documented in Utah, USA, from 2000 to 2003 (Carril et al. 2018), 25 were documented in Montana, USA, from 2013 to 2015 (Delphia et al. 2019), and 86 were added to the IUCN checklist of European Bees in 2017 (Rasmont et al. 2017). While efforts to better document bee communities are underway, we are simultaneously losing species due to anthropogenic mass extinction (Ceballos et al. 2015). An estimated 33% of all vertebrate species are considered threatened or endangered, with more than 300 species now extinct in the last ~520 yr (Lövei 2013, Miller 2013, Dirzo et al. 2014). Additionally, 40% of all invertebrate species are considered threatened, although this assessment is based on a mere 1% of the 1.4 million described invertebrate species (Dirzo et al. 2014), meaning that there are millions more left unassessed or even undescribed. Research has shown devastating population declines in some groups of bees, reaching as high as 96% (Cameron et al. 2011); however, without well-documented communities, it is difficult to understand the true conservation status of most bee species.

To address these knowledge gaps, there has recently been increased attention on the need to improve bee survey efforts, including how they are organized at national scales (Woodard et al. 2020, Staley et al. 2021), justifying (Breeze et al. 2021) or even reducing (Hegland et al. 2010) the cost- and labor-intensiveness of such surveys, and designing them to best meet research and conservation goals (Roy et al. 2016, Garratt et al. 2019). Surveys require considerable resources and infrastructure, as evidenced by the recent increase in citizen science (Parrish 2022) and reliance on high-throughput methods, such as automated monitoring and eDNA (Barlow and O’Neill 2020). Moreover, there are tremendous inconsistencies among studies aimed at quantifying bee communities; studies vary, among other factors, in the number of sites, sampling effort, and level of taxonomic identification. For example, Scheper et al. (2015) studied 64 sites but only 2 sampling trips per year, whereas Williams et al. (2015) investigated 9 sites with 8 sampling trips per year. It is unclear how these and other study design parameters might influence diversity measures, what portion of the bee community is documented, and how many resources are needed to best describe a given bee community. Therefore, it would be helpful to place any future study of native bee monitoring into a broader logistical context.

Here, we analyzed the existing research literature on bee surveys in and around agricultural lands to better understand how sampling methods affect 2 key measures of pollinator communities—bee abundance (“Abundance”) and diversity, using species richness (“Richness”) as a proxy. We evaluated this through 4 research objectives: summarizing *Study Design Parameters* from the literature, using *Abundance and Richness Curves* to understand how these measures are related, evaluating the *Impacts of Study Design Parameters on Community Measures*, and discussing some common ways for *Describing Bee Communities*. Our purpose is to determine the relative distributions of key aspects of previous experimental designs to inform and ideally optimize future survey efforts.

This review of the literature focuses on evaluating bee communities in agricultural settings because the ultimate benefit of their ecosystem service is through crop pollination and increased seed and fruit set to the majority of the world’s most important crops (Khalifa et al. 2021). These pollination services provide us with important vitamins and nutrients for the human diet (Lorenzo-Felipe et al. 2020). However, while bee populations are declining, agricultural reliance on pollination services is increasing (Aizen et al. 2008, 2019, Lautenbach et al. 2012, Koh et al. 2016). Therefore, conservation efforts to support bee communities in agricultural areas are occurring worldwide, emphasizing a need to understand how to design surveys to best document these communities.

While abundance and richness were the most commonly used and reported community measures in our corpus, it is worth noting that there are other community measures used in this area of research such as evenness, Shannon’s index (Shannon and Weaver 1964), and Simpson’s index (Simpson 1949). Species richness (*S*) is often an imperfect descriptor of a community since it does not incorporate variation among species in its measure. For example, for a given abundance of 100 specimens in a simple community of 2 species, an equal evenness (50 bees of each species) results in an effective species number of *N*_*e*_ = 2.00, whereas a highly skewed distribution (e.g., 99 of one species and 1 of the other) is effectively a community composed of a single species (*N*_*e*_ = 1.02). Moreover, any survey effort and sampling statistic are subject to certain biases, such as nonsampling error (sample abundance is insufficient to detect a given species, especially rare ones) and unevenness (skew among the representative species). A standard quantification of species richness that takes these factors into account is the Shannon’s index, *H*, which is the negative sum of the products of the proportion of each species with the log of that proportion (Supplementary Table S1). While a standard calculation in community ecology (Hurlbert 1971, Dejong 1975), the index is unitless and assumes all species in a community have been sampled. Other measures, such as effective species number, could be more intuitive (Jost 2006, Chao and Jost 2012). Evenness is then calculated as the ratio of effective versus actual species number, where for equally proportional species *E* = 1.00 and highly skewed communities approach 0.00. Alternatively, Simpson’s index calculates the effective species number as the inverse of the sum of squared proportions (Supplementary Table S1). Nielsen et al. (2003) demonstrated that the Simpson’s index (*D*) can be conservative because it is highly subject to nonsampling error at low abundances and thus proposes an unbiased estimator (Supplementary Table S1). To put these considerations into practice, we use a sample dataset (Levenson and Tarpy 2023) to describe the relative relationships among measures and provide insights into a standard means of reporting among bee studies.

## Methods

### Study Selection

Publications were collected from Web of Science, Mendeley, and Google Scholar from January 2018 through August 2022 using a series of 3 criteria screenings, conducted manually. The first screening involved searching for keywords from 2 lists in the title, abstract, or both. Publications could have included multiple keywords from each list. Several variations of keywords were considered simultaneously (i.e., including an “*” at the end of the search root). Keyword List 1 generally referred to “pollinators” and included pollinat*, bee*, wild bee*, native bee* visitor*, communit*, species, abundance, richness, insect*, service*, and pollination service*. Keyword List 2 generally referred to “landscape” and included landscape*, habitat*, seminatural, hedgerow*, floral resource*, flower*, wildflower*, strips, planting*, agricultur*, agro-ecosystem, agri-environment*, farm*, crop, yield, ecosystem, and conservation.

During the second screening, we only considered studies that were (i) published by 31 August 2020 (ii) as primary, empirical research; (iii) were conducted in agricultural landscapes; and (iv) examined bee communities (v) as a whole. Studies that researched a focal species or genus were excluded since the purpose was to focus on methods describing entire bee communities.

For the third and final screening, studies were included in our final corpus if reporting for our study design parameters of interest (described below) was readily available from the main text of the publication or easily accessible in Supplementary Material.

### Study Design Parameter Documentation

We manually extracted study design parameters of interest from the studies that met our preceding criteria (Table 1). We first documented the “Start year of study,” “Country of study,” “Continent of study,” and “State” (if conducted in the United States). We then documented the “Study duration” (in years), the “Number of sites” visited, the number of “Sampling trips per year,” and the “Sample time” per trip. “Site” was defined by the authors of individual studies. If any of these measures varied across multiple years of a given project, we calculated the mean. Next, we documented the “Collection method” used and “Level of identification.” “Collection method” categories included sweep net (“Net”), visual identification (“Visual”), pan trap (“Pan”), or some combination of these methods. While there were a small number of studies that used other sampling methods (e.g., blue vane traps), the number of such studies that fit our criteria was too low for analysis thus we limited the collection methods considered for inclusion to those listed above. “Level of identification” categories included “Species,” “Genus,” “Morphospecies,” or “Family.” Our study design parameters of interest were documented as they are critical components of study design when researching bee communities but differ greatly across studies, and so our objective is to determine the extent to which they may impact community measures. We then documented bee “Abundance” (the total number of specimens) and “Richness” (the total number of taxa collected). These 2 community measures were selected as they are the most commonly used and reported in the screened papers. Some papers collected data in multiple locations or using multiple sampling methods. When the information was provided separately in the original publication or supplemental material for each of these factors, we analyzed the data separately.

**Table 1.** Variables, and their descriptions, used during analyses

Variable	Description
“Continent of study”	Continent on which the study was conducted
“Country of study”	Reported as individual countries wherever possible
“State”	United States only; reported as individual states whenever possible
“Start year of study”	First sampling year reported
“Study duration”	Range (in years) of sampling years reported
“Number of sites”	Site collected; averaged over the collection years
“Sampling trips per year”	Sampling trips reported; averaged over the collection years
“Collection method”	Netting, pan trap, visual identification, and combinations
“Sampling time”	Averaged by sample trip; split by method whenever separate independent variables reported
“Level of identification”	Species, genus, morphospecies, and family; analyzed as an ordinal measure
“Abundance”	Total number of organisms identified, typically log-transformed for visualization and statistics, split by collection method whenever separately reported
“Richness”	Number of distinct taxa identified, split by collection method whenever separately reported

We also grouped studies into having 5 research goals: Impact of Practice on Bees, Impact of Landscape on Bees, Impact of Bees on Plants, Impact of Plants on Bees, and Other. Any given study could include multiple research topics, so these categories were not mutually exclusive. Those focusing on the Impact of Practice on Bees included the topics of crop vegetation, natural areas, hedgerows, flower strips, pesticide use, seed mix development for habitat plantings, installation of habitat plantings, or a combination of the above. Studies testing the Impact of Landscape on Bees included the topics of habitat types, landscape influence, or across large geographic distances. Studies focusing on the Impact of Bees on Plants included the topics of pollination impact on crop plants or noncrop plants. Studies covering the Impact of Plants on Bees included the topics of the influence of plant diversity on bees, the influence of bloom cover on bees, or some other pollination network measure. Finally, the Other category included the topics of bee functional diversity, nonbee insect sampling, bee health, and nesting resources. Studies that addressed multiple research goals were counted in each category they addressed. If a study collected data on multiple topics within a research goal, this was documented; however, we calculated each study as only contributing one instance to each goal (i.e., we did not calculate additively).

### Statistical Analysis

All statistics were performed in R 4.2.2–1.79 High Sierra Build (8160) (R Core Team 2023). Data were imported and organized using functions from the “readxl” (Wickham et al. 2023) and “tidyverse” (Wickham et al. 2019) packages. Data from statistical tests were processed using functions from the “broom” package (Robinson et al. 2023). All tests were performed with a significance of α = 0.05 with 2-tailed tests. Where appropriate, medians are reported with interquartile ranges.

### Study Design Parameters

Study design parameter distributions were assessed for normality using Shapiro–Wilk tests using the Shapiro.test() function from the “stats” package (R Core Team 2023). To compare the relationship between “Study duration” and “Start year of study,” Spearman’s ranked correlations were performed using the rcorr() function from the “Hmisc” package (Harrell and Dupont 2023). Spearman’s ranked correlations were performed because this method assumes only a monotonic relationship between variables rather than a strictly linear relationship. “Collection method” and “Sampling time” were compared using Kruskal–Wallis with pairwise Wilcoxon sign tests with a Benjamini–Hochberg correction for post hoc tests. Visualizations were created using ggplot2() with marginal histograms generated using ggMarginal() from the “ggExtra” package (Attali and Baker 2023). Significance groups were visualized using multcompLetters4() from the “multcompView” package (Graves et al. 2023). Multipart figures were created using ggarrange() from the “ggpubr” package (Kassambara 2023). Colors were chosen from palettes generated by the “khroma” package (Frerebeau 2023).

### Abundance and Richness Curves

“Abundance” and “Richness” distributions were tested with Shapiro–Wilk tests as described above. We used the nls() function to assess the Abundance–Richness curve and conduct model comparisons, with parameters for the exponential function initially estimated manually (Desmos 2023) to *b* = 100 and *k* = 0.0002. We assessed a nonlinear model for fit using functions from the “nlstools” package (Baty et al. 2015). Residuals were assessed with nlsResiduals(). Outliers were identified using jackknife and leave-one-out resampling with nlsJack(). Confidence intervals were estimated with nlsBoot() and nlsBootPredict(). We generated a log–log model using the lm() function. Visualizations were conducted as described above.

### Impacts of Study Design Parameters on Community Measures

**Fig. 1.**
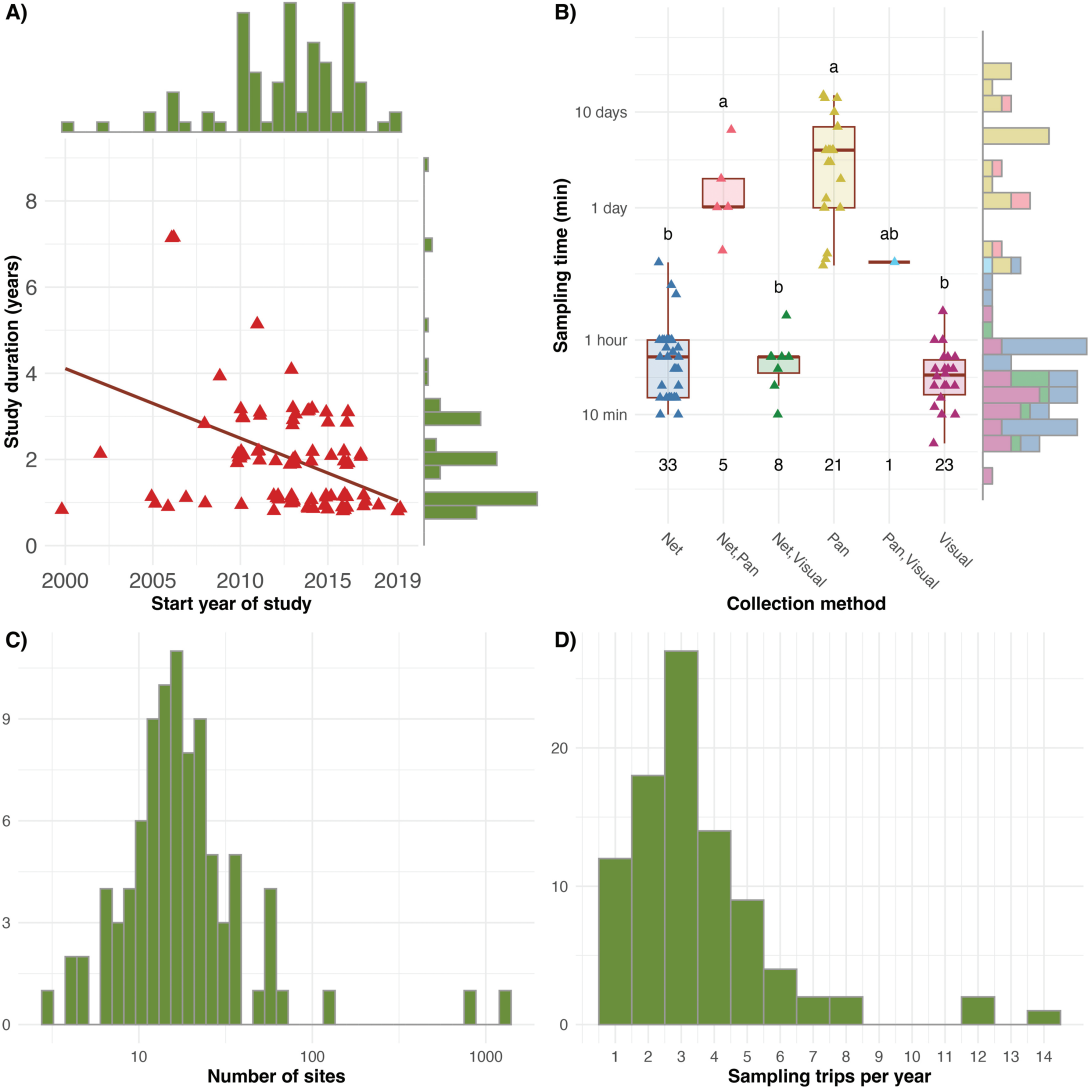
(A) Interaction between “Start year of study” (median = 2013; IQR = 5) and “Study duration” (median = 2, IQR = 2), indicating a negative correlation (Rho = −0.31; *P* = 0.0025) between the two with marginal histograms, illustrating the distributions of each. (B) Interaction between “Sampling time” and “Collection method,” demonstrating that sampling time for pan traps is on the order of days (median = 3 days; IQR = 4.26 days), while other methods are on the order of minutes (median = 30 min; IQR = 40 min), with a marginal histogram illustrating the bimodal distribution of sampling time. (C) Histogram illustrating the approximately logarithmic distribution of the “Number of sites” (median = 16; IQR = 12). (D) Histogram showing the distribution of the number of “Sampling trips per year” (median = 3; IQR = 2).

Based on visual assessments (Fig. 1), “Study duration,” “Sampling time,” “Number of sites,” and “Sampling trips per year” were log-transformed to limit skew. Categorical study design parameters with multiple levels were encoded with each level as a separate binary “dummy” variable. For example, “Collection method” was encoded as 3 separate binary variables, one for each collection method (“Net,” “Visual,” and “Pan”). Those studies that used multiple methods but reported a single measure of “Abundance” and “Richness” (*n* = 14) were considered positive for each method they used. Study design parameters were standardized for analyses. Regression modeling was conducted using the lm() function with residual diagnostics conducted using Shapiro–Wilk test as described above, the leverage of individual datapoints was assessed visually. Variables were selected using the stepAIC() function from the “MASS” package (Venables and Ripley 2002) using forward–reverse selection. Regression models were compared using the analysis of deviance test with anova(). Additional visualization and reporting were conducted using sum() and plot_summs() from the “jtools” package (Long 2022).

To analyze the impacts of study design parameters on “Abundance,” we conducted initial model testing with “Start year of study,” “Study duration,” “Collection method,” “Sampling time,” “Number of sites,” and “Sampling trips per year” as predictor variables and “Abundance” log-transformed as the dependent variable. A priori interaction terms between “Start year of study” and “Study duration,” and “Collection method” and “Sampling time” parameters were also included. This model was used to initialize the stepwise regression.

To analyze the impacts of study design parameters on “Richness,” we generated an initial model using the residuals of “Richness” calculated from an asymptotic model including “Abundance” as described above. We tested these residuals against the study design parameters “Start year of study,” “Study duration,” “Collection method,” “Sampling time,” “Number of sites,” “Sampling trips per year,” and “Level of identification.” We also included the interaction terms between “Start year of study” and “Study duration,” and “Collection method” and “Sampling time.”

### Describing Bee Communities

To describe the relative relationships among 3 measures of community diversity—Shannon’s, Simpson’s, and Nielsen’s indexes—we used a sample dataset (Levenson and Tarpy 2023) to describe the relative relationships among measures. We calculated each of these 3 estimates of community richness for each location (*n* = 16) in each year (*n* = 3). We then standardized the measures by relativizing each to the mean and SD of each calculation and then regressed against both the abundance (natural log-transformed) and richness of the sample dataset.

## Results

The broad screening of publications using our keywords resulted in 354 studies, but further refinement by publication date resulted in an initial set of 179 studies. Implementing our screening criteria (see above) resulted in a final corpus of 72 studies for analysis, spanning 20 yr of publication (Table 2 and Supplementary Table S2). Three of the studies were published after August 2020 (Levenson and Tarpy 2022, 2023, Levenson et al. 2022) but were still included because of our ready access to their entire datasets. The main reason studies were excluded from our final corpus because (i) a variable of interest was not reported or easily accessible (43.6%); (ii) the study was not primary, empirical research (30%); or (iii) the study did not consider the bee community as a whole (12%). The remaining 14.5% of excluded publications were for multiple reasons (Supplementary Table S3).

**Table 2.** Final corpus of studies used during analysis. For full reporting of variables, see Supplementary Table S1

Citation	Location	Citation	Location
Klein et al. (2003)	Indonesia	Macdonald et al. (2018)	New Zealand
Hannon and Sisk (2009	Arizona, USA	Tangtorwongsakul et al. (2018)	Thailand
Hagen and Kraemer (2010)	Kenya	Tucker and Rehan (2018)	New Hampshire, USA
Jha and Vandermeer (2010)	Mexico	Ouvrard et al. (2018)	Belgium
Blaauw and Isaacs (2014)	Michigan, USA	Hipólito et al. (2018)	Brazil
M’Gonigle et al. (2015)	California, USA	Hass et al. (2018)	France, Germany, Spain, UK
Nayak et al. (2015)	UK	Grab et al. (2018)	USA
Grass et al. (2016)	Germany	Wood et al. (2018)	Michigan, USA
Zou et al. (2017)	China	Bartual et al. (2018)	Italy
Kremen et al. (2018)	California, USA	Kremen et al. (2018)	California, USA
Pfiffner et al. (2018)	Switzerland	Breland et al. (2018)	South Carolina, USA
Klein et al. (2012)	California, USA	Happe et al. (2018)	Germany
Rollin et al. (2013)	France	Beduschi et al. (2018)	Germany
Benjamin et al. (2014)	USA	Kratschmer et al. (2018)	Austria
Cariveau et al. (2013)	New Jersey and California, USA	Ganser et al. (2018)	Switzerland
Le Féon et al. (2013)	France	Mallinger et al. (2019)	North Dakota, USA
Bailey et al. (2014)	France	Fijen et al. (2019)	Italy
Hopfenmüller et al. (2014)	Germany	Castle et al. (2019)	Germany
Riedinger et al. (2015)	Germany	Karamaouna et al. (2019)	Greece
Connelly et al. (2015)	New York, USA	Sirami et al. (2019)	France, UK, Germany, Spain, Canada
Scheper et al. (2015)	Germany, Sweden, Netherlands, UK	Xie et al. (2019)	China
Holland et al. (2015)	England	Toivonen et al. (2019)	Finland
Williams et al. (2015)	Florida, Michigan, and California, USA	Cunningham-Minnick et al. (2019)	Ohio, USA
Kremen and M’Gonigle (2015)	California, USA	Eeraerts et al. (2019)	Belgium
Mallinger et al. (2016)	Wisconsin, USA	Kratschmer et al. (2019)	Spain, France, Austria, Romania
Siregar et al. (2016)	Sumatra	Krimmer et al. (2019)	Germany
Blitzer et al. (2016)	New York, USA	Riojas-López et al. (2019)	Mexico
Harrison et al. (2019)	New Jersey, New York, and Pennsylvania, USA	Sanchez et al. (2020)	Spain
Gervais et al. (2017)	Canada	Brown et al. (2020)	Australia
Sutter et al. (2017)	Switzerland	Du Clos et al. (2020)	Maine, USA
Bukovinszky et al. (2017)	Netherlands	Jovani et al. (2020)	Mexico
Nicholson et al. (2017)	Vermont, USA	MacInnis et al. (2020)	Canada
Eeraerts et al. (2017)	Belgium	McKerchar et al. (2020)	UK
Morrison et al. (2017)	Spain	Levenson and Tarpy (2023)	North Carolina, USA
Warzecha et al. (2018)	Germany	Levenson and Tarpy (2022)	North Carolina, USA
Martins et al. (2018)	Canada	Levenson et al. (2022)	North Carolina, USA

Most studies incorporated more than one research goal, the most common of which was Impact of Landscape on Bees (70 studies, 97.2%) followed by the Impact of Plant on Bees (61 studies, 84.7%), then the Impact of Practice on Bees (47 studies, 65.3%), Other (46 studies, 63.9%), and finally the Impact of Bees on Plants (32 studies, 44.4%). The 3 most commonly researched topics were influence of bloom cover on bees (55 studies, 76.4%), landscape influence (44 studies, 61.1%), and influence of plant diversity on bees (41 studies, 56.9%). The 3 least commonly researched topics were pesticide use (2 studies, 2.8%), impact on noncrop plants (2 studies, 2.8%), and bee health (1 study, 1.4%).

**Fig. 2.**
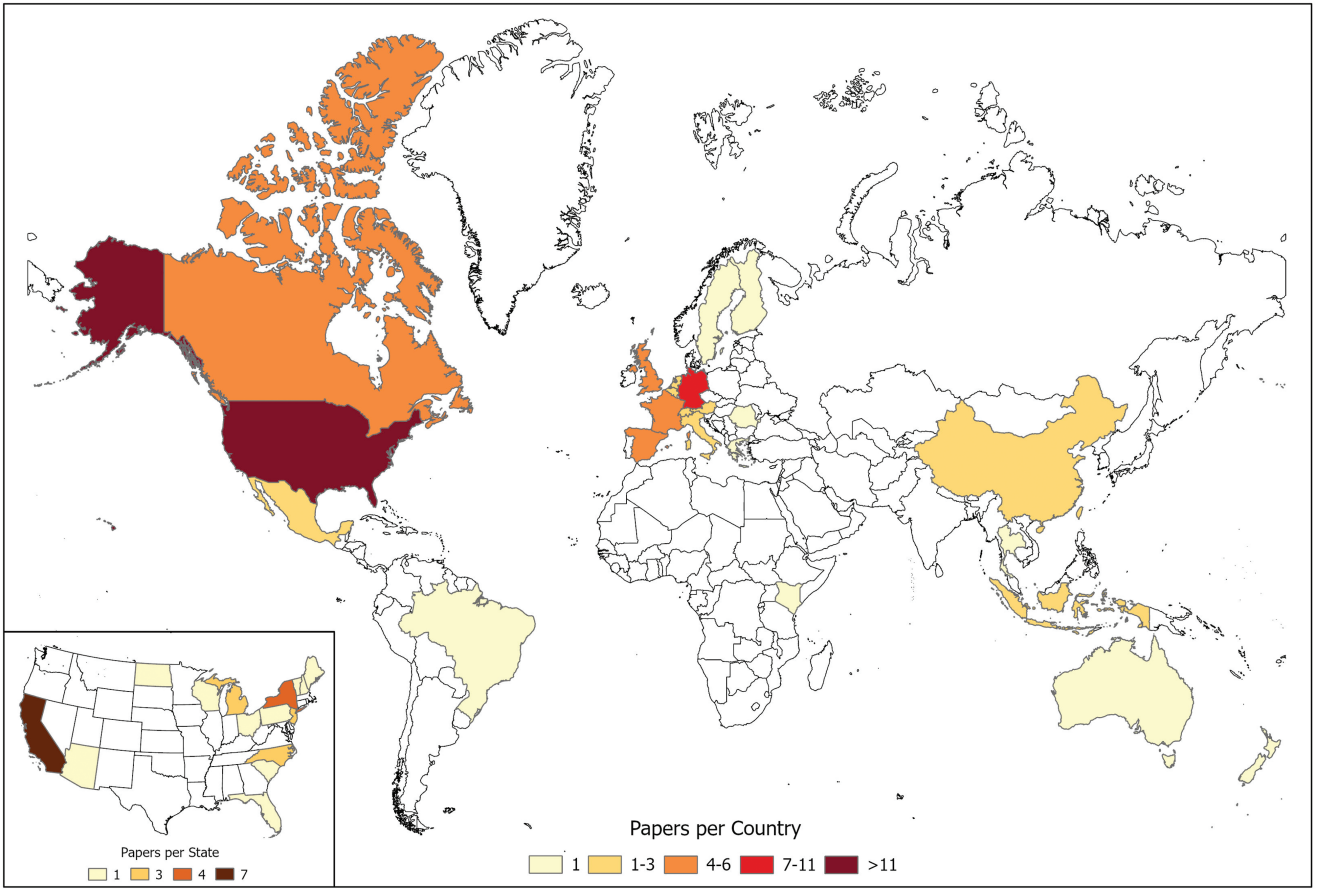
Map showing the distribution of studies conducted in each country. The inset map shows the distribution of studies conducted in each state when the study was conducted in the United States. Thank you to James Goethe for visualizing this figure in ArcGIS (version 2.9, Environmental Systems Research Institute, Redlands, CA).

The studies included in our corpus spanned 24 countries (Fig. 2) but were overwhelmingly biased to Europe (39 studies, 54.2%) and North America (42 studies, 58.3%). Those conducted in the United States (34 studies, 47.2%), Germany (9 studies, 12.5%), France, Spain, and Canada (all with 5 studies, 6.9% each) were the most common countries. Within the United States, only 15 of the 50 states were represented. Within the United States, there was again a geographic bias in study selection, with California being the most highly represented (7 studies, 20.6%).

### Study Design Parameters

Projects selected for analysis were initiated between 2000 and 2019 (Fig. 1A). Neither “Start year of study” (*W* = 0.938, *P* < 0.0001) nor “Study duration” (*W* = 0.666, *P* < 0.0001) were normally distributed, with more studies found in more recent years (median = 2013, IQR = 5). The majority of studies lasted a single year (41 studies out of *n* = 91, 45.1%, median = 2, IQR = 2), with only 25% of studies lasting 4 yr or longer. Log-transformation did not improve the distribution of either of these parameters (*P* < 0.0001), leading to concerns of bias in the final models such that older studies would naturally progress for longer durations. Indeed, we found a significant negative correlation between “Start year of study” and “Study duration” (Rho = −0.31, *P* = 0.0025; Fig. 1A).

The most commonly used “Collection method” was “Net” (33 studies, 36.3%), followed by “Visual” (23 studies, 25.3%), then “Pan” (21 studies, 23.1%), and finally some combination of the three (14 studies, 15.4%). “Sampling time” was bimodal and non-normally distributed (*W* = 0.471, *P* < 0.0001), owing to the vast differences in sampling times among studies that used the “Pan” sampling method (median = 3 days, IQR = 4.26 days) and those that did not (median = 30 min, IQR = 40 min). “Sampling time” did differ significantly by “Collection method” (χ^2^_5_ = 58.25; *P* < 0.0001), with those studies using the “Pan” sampling method having a much longer “Sampling time” than those that did not (Fig. 1B).

The study design parameter “Number of sites” was not normally distributed (*W* = 0.19, *P* < 0.0001, median = 16, IQR = 12), with 2 extreme outlier studies reporting 812 (Rollin et al. 2013) and 1,305 (Sirami et al. 2019). Excluding these studies and log transforming the data resulted in a normal distribution (*W* = 0.986, *P* = 0.436), without changing the median or IQR (Fig. 1C). The study design parameter “Sampling trips per year” was also not normally distributed (*W* = 0.79, *P* < 0.0001, median = 3, IQR = 2), with 3 being the most common number of trips conducted (24 studies, 26.4%) and a maximum of 14 trips per year (Tucker and Rehan 2018; Fig. 1D).

It would be expected that studies that identify to a more specific taxonomic level (and so would have more taxonomic units) would report higher “Richness” compared with those that only reported using a higher taxonomic level (and so would have fewer taxonomic units). We therefore sought to observe bias in the “Level of identification.” We took the nonstandard “Morphospecies” identification to be approximately at the level of “Genus” for ordinal analyses. Most studies identified to “Species” (47 studies, 51.6%), then “Genus” (23 studies, 25.3%), then “Morphospecies” (19 studies, 4.4%), and finally “Family” (2 studies, 2.2%).

When considering all of the study design parameters from our corpus, designing a study to survey bees in agroecosystems that meets the 50th percentile would include collecting at 16 sites across 2 yr with 3 trips per year using sweep nets for 30 min (Table 3). A study that meets the 95th percentile would include collecting at 58 sites across 5 yr with 8 trips per year (Table 3).

**Table 3.** Median (50th) and 95th percentiles of studies assessed in these analyses for each study parameter observed. “Collection method” and “Level of identification” are reported as proportion of studies using each category inclusive of those studies using multiple categories

	Quantile		
Parameter	50%	95%	%Studies
“Abundance” (specimen)	2,094	11,711	
“Richness” (taxa)	53	158	
“Start year of study”	2,013	2,017	
“Study duration” (years)	2	5	
“Number of sites”	16	58	
“Sampling trips per year”	3	8	
“Sampling time”			
Non-pan collected (minutes)	30	119	
Pan collected (days)	3	14	
“Collection method”			
Net			51%
Visual			35%
Pan			30%
“Level of identification”			
Species			52%
Genus			25%
Morphospecies			21%
Family			2%

### Abundance and Richness Curves

Without access to underlying local community information, and acknowledging that some communities are inherently richer in taxa than others, we estimate that the 50th percentile of “Richness” in the meta-community of this analysis is 72 species with 3,881 specimens needed to reach that target, while to estimate 95% of this meta-community’s “Richness” (estimated at 136 species), 16,773 specimens are required. While the association is significant, the residual error around the Abundance–Richness curve remains high, suggesting that collecting a greater number of insects is not the sole factor in increasing the number of taxa (see below).

**Fig. 3.**
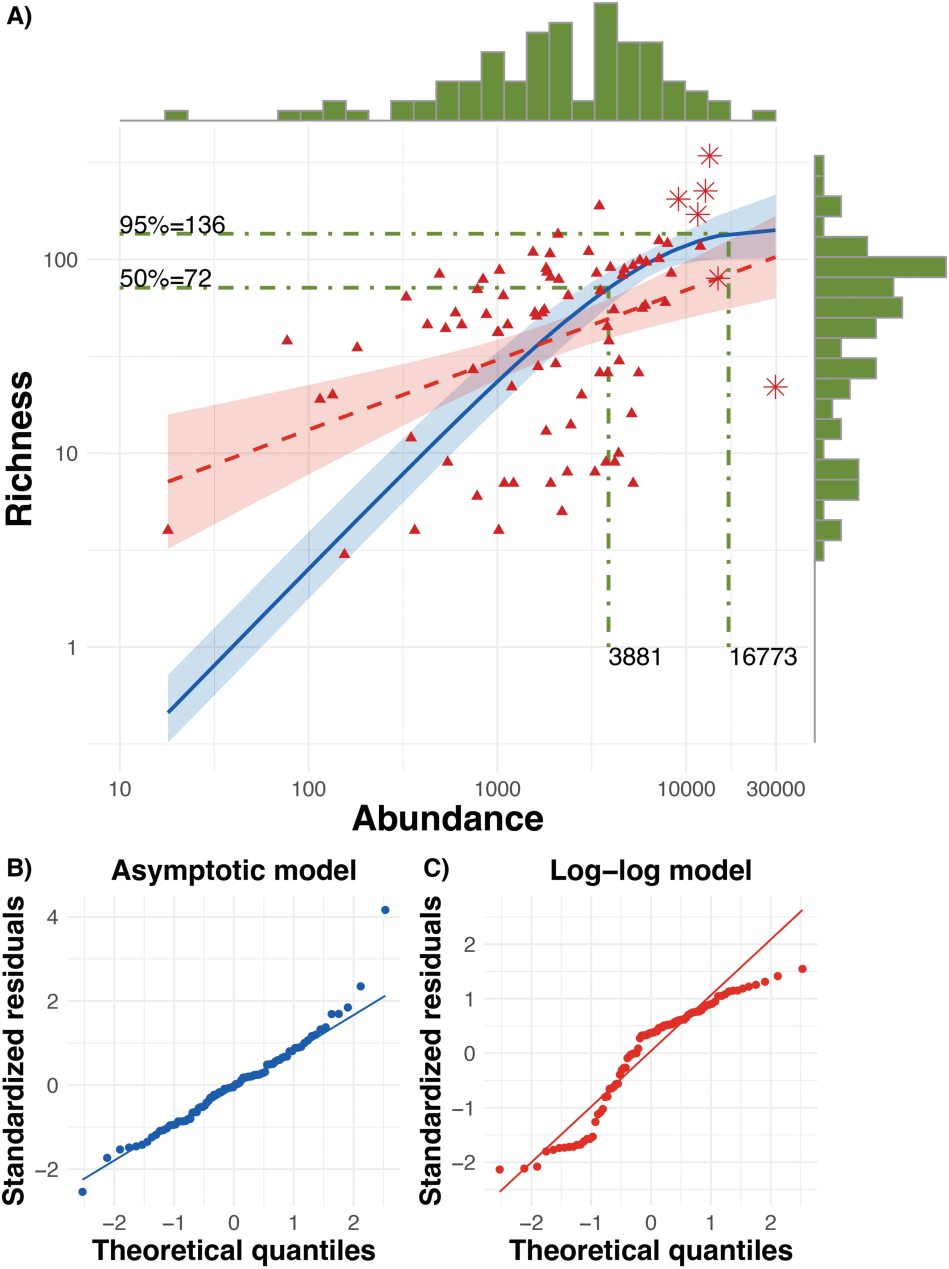
(A) Scatterplot and line representing the statistical relationship between “Richness” and “Abundance.” The asymptotic model *y* = b*(1 − e^(−*kx*)^) is represented by the solid line. The band around this line represents bootstrapped estimated 2.5th and 97.5th percentile confidence intervals. The log–log model is represented by the dashed, line with the band around it representing confidence intervals. Asterisk points represent statistical outliers in the model that exert influence on the maximum (*b*) and rate of increase (*k*). The 2 dotted lines illustrate the “Abundance” at 50% (72) and 95% (136) cutoffs of total “Richness.” Marginal histograms present the distribution of the data. Both axes are shown on a log scale. (B) QQ plot for the asymptotic model. (C) QQ plot for the log–log model.

Neither “Abundance” (*W* = 0.705, *P* < 0.0001) nor “Richness” (*W* = 0.822, *P* < 0.0001) was normally distributed and was instead strongly right-skewed. Log-transformation did not eliminate the skew (“Abundance”: *W* = 0.925, *P* < 0.0001; “Richness”: *W* = 0.944, *P* = 0.0008), though they were visually closer to a normal distribution; distributions are presented as marginal plots in Fig. 3A. In order to address this concern and to test if the relationship between “Abundance” (*x*) and “Richness” (*y*) was asymptotic, we tested a negative exponential model with the following formula: $y=b*(1-e^{-kx})$ and compared this to a log–log model as the appropriate nonasymptotic model. The asymptotic model was significant with both coefficients in the formula significant. The maximum (*b*) was 143 ± 2.90 (*t* = 4.94, *P* < 0.0001), and the rate of increase (*k*) was 1.786*10^−4^ ± 0.650*10^−4^ (*t* = 2.75, *P* = 0.007). Residuals were not normally distributed (*W* = 0.960, *P* = 0.008), suggesting that the model was not entirely unbiased to the fit (solid, blue line in Fig. 3A). The log–log model was similarly significant, albeit with a relatively weak fit (*F*_1,86_ = 18.53; *P* < 0.0001; *R*^2^ = 0.17; dashed, red line in Fig. 3). Residuals were again not normally distributed (*W* = 0.91, *P* < 0.0001). Q–Q plot comparisons suggested that the exponential model was a better fit (Fig. 3B and C) and to get a numerical understanding of this, we generated predicted and residual values for both the asymptotic and the log–log model. We back-transformed the predicted and residual values to raw “Richness,” so that both models were on the same scale.

We calculated a residual standard error using the formula: $RSE=\surd \sum {\left(y-\hat{y}\right)}^{2}/df$ where *y* = “Richness” and ŷ = the predicted “Richness” of each model. The RSE for the asymptotic model was 49.71 (df = 86) and 53.58 (df = 86) for the log–log model, suggesting a slight improvement in fit for the asymptotic model. In further exploring the asymptotic model, the leave-one-out resampling identified 6 observations as potential outliers representing the largest studies, all with “Abundances” over 9,000. This is somewhat expected as there are fewer studies at this scale compared to smaller scales in our corpus; however, these large studies represent the region of interest in resolving an asymptotic maximum. Therefore, despite the influence of these studies, we kept them in the model (marked with asterisks in Fig. 3A).

### Impacts of Study Design Parameters on Community Measures

**Fig. 4.**
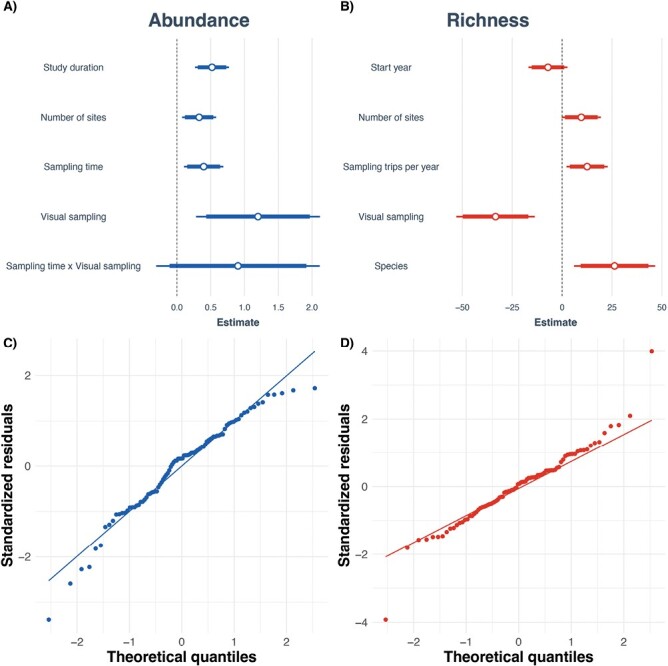
Regression results of the impact of study design features on “Abundance” (A, C) and “Richness” (B, D). The means and confidence intervals (0.95 for the thin bars, 0.90 for the thicker bars) of each study parameter included in the “Abundance” model (A) and the “Richness” model (B). QQ plots for the “Abundance” model (C) and “Richness” model (D), illustrating the broad goodness of fit with some outliers.

In analyzing the impacts of study design parameters on “Abundance,” we found the stepwise regression to be significant (*F*_5,84_ = 6.38, *P* < 0.0001, *R*^2^ = 0.23), but it did not significantly differ compared to the more complex model which included all terms (*F*_7,77_ = 0.64, *P* = 0.72). This simplified model (resulting model after stepwise regression) included a set of 5 terms (Fig. 4A) with the coefficients for “Study duration” (*t* = 4.099, *P* < 0.0001), “Number of sites” (*t* = 2.596, *P* = 0.11), “Sampling time” (*t* = 2.703, *P* = 0.008), and the “Visual” level of “Collection method” (*t* = 2.604, *P* = 0.011) all identified as significant from zero, and an interaction between “Sampling time” and “Visual” included but nonsignificant from zero (*t* = 1.484, *P* = 0.142). A full list of coefficients is reported in Table 4. The residuals were not normally distributed (*W* = 0.964, *P* = 0.014), but the Q–Q plots showed no appreciable pattern (Fig. 4C).

**Table 4.** Regression results of the impact of study design parameters on “Abundance.” Coefficients are returned as mean centered and scaled to 1 SD. Means and standard errors of each coefficient are reported

	Abundance	
Study duration	0.52***	(0.13)
Number of sites	0.33*	(0.13)
Sampling time	0.40**	(0.15)
Visual sampling	1.20*	(0.46)
Sampling time × Visual sampling	0.90	(0.61)
*N*	90	
*R* ^2^	0.28	

All continuous predictors are mean centered and scaled by 1 SD. The outcome variable is in its original units.

***P < 0.001;

**P < 0.01;

*P < 0.05.

In analyzing the impacts of study design parameters on “Richness,” we found the final stepwise model was significant (*F*_5,82_ = 6.350, *P* < 0.0001, *R*^2^ = 0.235), but it did not significantly differ from the initial, more complex model, which included all terms (*F*_10,82_ = 0.465, *P* = 0.907). This simplified model (a resulting model after stepwise regression) included a set of 5 terms with “Sampling trips per year” (*t* = 2.418, *P* = 0.018), the “Visual” level of “Collection method” (*t* = −3.378, *P* = 0.001), and the “Species” level of “Level of identification” (*t* = 2.573, *P* = 0.012) all significant. “Start year of study” (*t* = −1.442, *P* = 0.153) and “Number of sites” (*t* = 1.957, *P* = 0.054) were included in the model but were not different from zero (Fig. 4B). A full list of coefficients is reported in Table 5. The residuals were normally distributed (*W* = 0.976, *P* = 0.110), and the Q–Q plot showed no apparent pattern (Fig. 4D). Visual inspection of the outliers identified 2 studies (Rollin et al. 2013, Sirami et al. 2019) that were exerting extreme leverage on the model. These correspond to the studies with a large “Number of sites” mentioned above. Removing these studies from the analysis results in a similarly significant final model (*F*_6,79_ = 5.317, *P* < 0.0001, *R*^2^ = 0.2.40) that did not materially change the significances of the parameters “Sampling trips per year” (*t* = 2.483, *P* = 0.015), the “Visual” level of “Collection method” (*t* = −3.985, *P* = 0.001), the “Species” level of “Level of identification” (*t* = 2.239, *P* = 0.028), “Start year of study” (*t* = −1.520, *P* = 0.133), or “Number of sites” (*t* = 1.267, *P* = 0.209).

**Table 5.** Regression results of the impact of study design parameters on “Richness.” Coefficients are returned as mean centered and scaled to 1 SD. Means and standard errors of each coefficient are reported

	Richness	
Start year	−7.07	(4.90)
Number of sites	9.62	(4.92)
Sampling trips per year	12.53*	(5.18)
Visual sampling	−33.33**	(9.87)
Species	26.27*	(10.21)
*N*	88	
*R* ^2^	0.28	

All continuous predictors are mean-centered and scaled by 1 SD. The outcome variable is in its original units.

**P < 0.01;

*P < 0.05.

### Describing Bee Communities

**Fig. 5.**
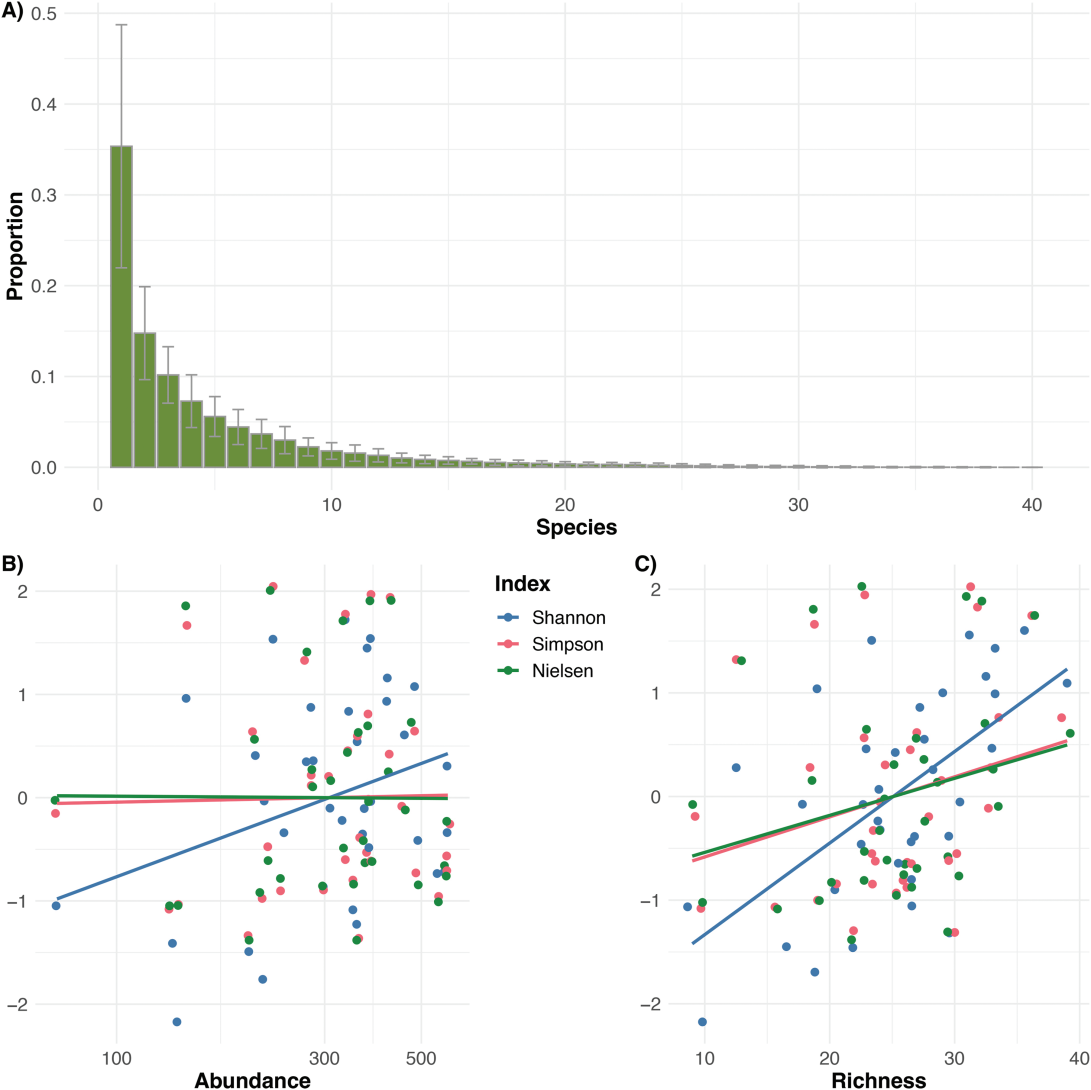
Unevenness of ranked species (A) as well as diversity indices as a function of “Abundance” (B) and “Richness” (C). Calculations of Shannon’s index (blue), Simpson’s index (red), and Nielsen’s index (green) were calculated for each of the 16 sampling sites across 3 yr (total = 48) in Levenson and Tarpy (2023). Because the different indices have different units, they were standardized to their respective means and SD and then plotted against the 2 main measures of community to illustrate trends. Shannon’s index is more sensitive to both “Abundance” and “Richness.” “Abundance” is visualized on a log scale for clarity.

Estimates of evenness among the yearly sampling sites in Levenson and Tarpy (2023) were relatively low with moderate variability (0.22 ± 0.019, range 0.09–0.59; Fig. 5A), verifying that the simple total number of species to quantify “Richness” may be missing important information about each sampled community. A comparative species accumulation curve shows that, even though it may be convention, Shannon’s index (*H*) tends to have numerically lower estimates of community diversity than Simpson’s index, albeit with lower variability. Moreover, since *H* assumes that all species in a community have been sampled, it is more sensitive to “Abundance,” especially at low sample size since the slope is marginally positive (*F*_1,34_ = 3.66, *P* = 0.064; Fig. 5B). In contrast, both Simpson’s (*D*) and Nielsen’s estimates of effective species number (*N*_*e*_) show equivalent measures of effective species number, at least at reasonable sample sizes (*n* > 50), but their estimates are more highly varied among samplings than *H*. However, even at finite abundances, their slopes are not significantly different from zero (both *F*_1,34_ < 0.011, *P* > 0.91), meaning that they provide reasonable estimates even at low to modest sample sizes. When comparing these estimators as a function of species richness *S*, Shannon’s is highly significantly associated (*F*_1,34_ = 18.7, *P* < 0.0001), with a much steeper slope (Fig. 5C), suggesting it is more sensitive than the other measures as more species are sampled in a community. Neither Simpson’s (*F*_1,34_ = 2.48, *P* = 0.12) nor Nielsen’s (*F*_1,34_ = 2.09, *P* = 0.16) showed a significant association with “Richness.”

## Discussion

Our meta-analysis describes the variation in sampling effort among various studies of bee communities in agroecosystems, and in doing so, it will help future researchers more readily gauge their expectations in developing experimental designs. Ultimately, it is more pertinent to look at “Abundance” and “Richness” in concert to address the question, “what are the most important study design parameters for documenting bee communities?” The most intuitively obvious answer, and easiest approach, is to collect more samples, although there are several factors that might work against that. First, in an ecological system with rare or even endangered species, collecting more bees may be difficult. Second, when working with cryptic or heterogeneously distributed species, naively collecting more specimens may simply increase the representation of the most common species. Finally, given the reality of human labor costs and limitations, balance must be maintained to efficiently collect to maximize the given result. Therefore, it is best to assess the causal factors that operate to increase “Abundance”—and thus “Richness”—and determine the best means to balance the benefits and costs of each of these factors.

In the major findings of our analysis, we show that study design parameters influence “Abundance” and “Richness.” We found that when designing a study, studies that exhibited higher “Abundance” tended to have a longer “Study duration” (in years), collected at more “Number of sites,” for a longer “Sampling time,” and used the “Visual” level of “Sampling method.” Controlling for “Abundance,” the study factors that positively influenced “Richness” are “Sampling trips per year” and the “Species” level of “Level of identification.” Of note, we found that the “Visual” level of “Collection method” negatively influenced “Richness.” These findings highlight areas where efforts in one study design parameter could be prioritized to address certain goals or to compensate for limitations in another parameter when designing future studies and developing standardized protocols. For example, if a project’s goal is to document bee richness in a specific region, our results suggest that increasing the temporal or seasonal spread of sampling effort (i.e., “Sampling trips per year”) is likely more important than increasing the “Number of sites,” at least within similar landscapes. Another consideration is that since the study design parameters “Sampling method” and “Level of identification” influenced “Abundance” and “Richness” differently, efforts in these parameters could be shifted depending on the goals of a specific project. In recent years, we have seen a decrease in “Study duration”; this could be driven by the bias of more-recent longer-term studies not being published or stem from reduced funding, labor difficulties, and a shift in focus away from community ecology research. The results from multiyear studies may also be impacted by the interannual changes that naturally occur in bee communities (Franzen and Nilsson 2013). Nonetheless, since “Study duration” was found to be an important parameter for quantifying “Abundance,” a clear conclusion of our meta-analysis is that descriptions of bee communities are more robust when conducted over 3 yr or more. In our analysis, we considered 3 common sampling methods: sweep netting (“Net”), visual identification (“Visual”), and pan trapping (“Pan”). It will be important when designing future studies to consider these sampling methods carefully as they were found to influence our key community measures, and each has its own benefits and drawbacks. While easy to implement, previous research has documented that pan traps can be influenced by surrounding landscapes (Kuhlman et al. 2021, Westerberg et al. 2021), disproportionately collect certain taxa within communities (Portman et al. 2020), collect different species than other sampling methods (Hutchinson et al. 2022)—especially small-bodied bee species (Pei et al. 2022)—and may not correlate to samples collected by other methods, even within the same community (Berglund and Milberg 2019, Hudson et al. 2020, Briggs et al. 2022). These limitations could mean pan traps are unreliable for certain research questions (Boyer et al. 2020). Net and visual sampling have their own drawbacks as well (McCravy 2018), including variation in net handling person-to-person and biases in data collection due to a person’s experience (Falk et al. 2019). However, our analysis was limited in that the only community diversity measure we analyzed was richness (due to low reporting of other diversity measures in our corpus). If one was to analyze another measure of the bee community, such as effective species number, the inferences concerning sampling method might be different. When deciding which sampling method to use in a sampling design, it is important to consider all factors and costs of each study’s individual characteristics, which include community assemblage of interest, the effectiveness of sampling methods, labor needs, and interest in nondestructive sampling (also see Droege et al. 2016).

With our analyzed literature, we show several knowledge gaps in research topics. For example, only 1 study focused on bee health (1.4%), 2 studies on pesticide use (2.8%), and 9 studies on nesting resources (12.5%). This highlights focal areas of future research as these are key aspects of bee conservation in agricultural areas. Some studies report collecting nonbee insects (23 studies, 31.9%), but only when this was a research question of the study. It is extremely likely that most projects collect nonbee insects, especially those utilizing passive sampling tools such as pan traps, but these samples were largely unreported and potentially discarded. This bycatch could provide important information on lesser studied insect species, and what to do with this bycatch is a discussion that should be had within this area of research. Indeed, there is increasing awareness that insect groups other than bees can be important pollinators of certain plants and crops (Orford et al. 2015).

Our analyses were limited by what was reported in the publications, which may not fully represent the true environmental factors or bee communities in each location. For example, we used the “Number of sites” as reported in each publication; however, people may define this variable differently. In some instances, sites were selected to be geographically distinct from each other at distances that would limit overlap in local bee populations. In other instances, site referred to a distinct sampling location that was within the same overall farm as another site. As researchers in this subject area work to improve and standardize bee monitoring efforts, we should be mindful to make terminology and reporting expectations clear and reproducible.

Improper reporting of study design elements was the main reason studies that were rejected from our final corpus (43.6%). This is concerning as the expectation for publication is that all methods and data needed to reproduce a study and its results are included at the time of publication. In the least, publication on bee surveys should include standard reporting of basic study design elements as well as abundance (*n*), species richness (*S*), and some measure of effective species number (*N*_*e*_) or diversity that incorporates evenness. Of the 72 publications that were analyzed, the majority reported species richness as their main description of the bee community, with very few reporting some other measures such as Shannon’s index, evenness, or others. In analyzing our sample dataset, we found Simpson’s or Nielsen’s estimators of effective species number were less conservative at lower abundance and richness measures compared to Shannon’s index. Comparing the trade-offs of different community measures (Hurlbert 1971, Dejong 1975) suggests that a discussion could be had as to how best to describe bee communities in future studies, especially with conservation goals in mind. One action that would easily allow further calculations of different community measures of any study would be the inclusion in supplemental material of a list of taxa and number of specimens collected at publication. Furthermore, as has previously been documented, information regarding identification materials used and specimen deposition is not always reported in publications (Montero-Castaño et al. 2022). As such, the validity of identifications and the use of specimens for future work are limited, especially if specimens are not deposited into long-term collections. Studies should strive to include this information during the publication process.

## Conclusions

There are still many knowledge gaps about studying and measuring bee communities that future research should work to address. We therefore conclude and recommend the following for future studies. (i) When conducting a study with a focus on other (nonagricultural) ecosystems, it will be important to develop context-specific recommendations, as our findings could be specific to agricultural areas. (ii) Studying each site more intensively over the course of the season is more important than increasing the number of sites to maximize estimates of species diversity, since it helps to capture different phenologies and species that are active at different times of the year. (iii) Studies that last 3 or more years have an outsized effect on maximizing estimates of abundance, so priority should be given to longer-term research. (iv) As is the standard in community ecology research, all studies of bee communities should not just report richness (total count of taxa found) but also evenness, effective species number, or ideally all three. We also recommend using the Simpson’s or Nielsen’s estimators over Shannon’s index since they are more intuitive and can be reported with variance estimates. Supplemental tables or public data with the abundances of each taxon will also be helpful for others to describe community composition. (v) Specimen identifications to species is a major logistical bottleneck. Lowering the barriers for identification of specimens to species would be one of the most effective ways of improving our descriptions of bee communities. Additionally, storing specimens properly and reporting their location in publications will be critical, so that specimens can be used in future work or reidentified if needed.

Our meta-analysis is a first step in contextualizing bee survey studies in agroecosystems to the greater literature with regard to key study design parameters. This information will help standardize methods among future studies and make it easier to optimize sampling efforts.

## Supplementary Material

saae001_suppl_Supplementary_Table_S1

saae001_suppl_Supplementary_Table_S2

saae001_suppl_Supplementary_Table_S3
