# Supplementary material for: Effects of study design parameters on estimates of bee abundance and richness in agroecosystems: a meta-analysis
Source: Ann Entomol Soc Am. 2024 Jan 19;117(2):92–106. doi: 10.1093/aesa/saae001 (PMC10933562; doi:10.1093/aesa/saae001)
Supplement: saae001_suppl_Supplementary_Table_S1 [file saae001_suppl_supplementary_table_s1.docx]

Effects of study design parameters on estimates of bee abundance and richness in agroecosystems: a meta-analysis

Hannah K. Levenson^1^*, Bradley N. Metz^2^, David R. Tarpy^2^

1. Department of Entomology and Plant Pathology, North Carolina State University, NC, USA

2. Department of Applied Ecology, North Carolina State University, NC, USA

*Corresponding Author: 2301 Gardner Hall, 100 Derieux Place, North Carolina State University, Raleigh NC, 27695; 919.434.7882; [hklevens@ncsu.edu](mailto:hklevens@ncsu.edu)

**Supp. Table 1**

| **Index** | **Variable** | **Equation** | **Reference** |
| --- | --- | --- | --- |
| Shannon’s | *H* | $-\sum_{i=1}^{S} p_{i}ln(p_{i})$ | Shannon and Weaver (1963) |
| Simpson | *D* | $\frac{1}{\sum_{i=1}^{S} p_{i}^{2}}$ | Simpson (1949) |
| Nielsen | *N_e_* | $\frac{{(n-1)}^{2}}{\sum_{i=1}^{S} p_{i}^{2}\left( n+1 \right)\left( n-2 \right)+3-n}$ | Nielsen et al. (2003) |
