# Supplementary material for: Effects of study design parameters on estimates of bee abundance and richness in agroecosystems: a meta-analysis
Source: Ann Entomol Soc Am. 2024 Jan 19;117(2):92–106. doi: 10.1093/aesa/saae001 (PMC10933562; doi:10.1093/aesa/saae001)
Supplement: saae001_suppl_Supplementary_Table_S2 [file saae001_suppl_supplementary_table_s2.docx]

Effects of study design parameters on estimates of bee abundance and richness in agroecosystems: a meta-analysis

Hannah K. Levenson^1^*, Bradley N. Metz^2^, David R. Tarpy^2^

1. Department of Entomology and Plant Pathology, North Carolina State University, NC, USA

2. Department of Applied Ecology, North Carolina State University, NC, USA

*Corresponding Author: 2301 Gardner Hall, 100 Derieux Place, North Carolina State University, Raleigh NC, 27695; 919.434.7882; [hklevens@ncsu.edu](mailto:hklevens@ncsu.edu)

**Supp. Table S2**. Table describing our corpus and corresponding variables of interest. When variables differed across years, we averaged the values for analysis. Collection method is abbreviated as: V = visual identification, N = sweep net, P = pan trap. Level of Identification is abbreviated as: F = family, G = genus, M = morphospecies, S = species.

| *Citation* | *Country* | *US State* | *Start Year* | *No. of Years* | *No. of Sites (Avg)* | *Sample Trips/Year (Avg)* | *Collection Method* | *Net Sample Time (min)* | *Pan Traps Sample Time (days; Avg)* | *Visual Sample Time (min)* | *Combined Method Sample Time* | *Net Abundance* | *Pan Traps Abundance* | *Visual Abundance* | *Combined Method Abundance* | *Net Species Richness* | *Pan Traps Species Richness* | *Visual Species Richness* | *Combined Method Species Richness* | *Level of Identification* | *Impact of Practice on Bees* | *Impact of Landscape on Bees* | *Impact of Bees on Plants* | *Impact of Plants on Bees* | *Other* |
| --- | --- | --- | --- | --- | --- | --- | --- | --- | --- | --- | --- | --- | --- | --- | --- | --- | --- | --- | --- | --- | --- | --- | --- | --- | --- |
| Klein et al. 2003 | Indonesia |  | 2000 | 1 | 24 | 3 | V, N | 5 |  | 25 |  |  |  |  | 2038 |  |  |  | 29 | G, S |  |  | Y | Y |  |
| Hannon and Sisk 2009 | USA | AZ | 2002 | 2 | 8 | 3 | V, N | 20 |  | 20 |  |  |  |  | 1902 |  |  |  | 107 | G, S |  | Y |  | Y |  |
| Hagen and Kraemer 2010 | Kenya |  | 2005 | 1 | 18 | 12 | V |  |  | 60 |  |  |  | 7968 |  |  |  | 121 |  | S |  | Y |  | Y |  |
| Jha and Vandermeer 2010 | Mexico |  | 2006 | 1 | 7 | 6 | P |  | 14 |  |  |  | 648 |  |  |  | 46 |  |  | G, M, S |  | Y |  | Y | Y |
| Blaauw and Isaacs 2014 | USA | MI | 2009 | 4 | 5 | 1 | V |  |  | 30 |  |  |  | 5240 |  |  |  | 7 |  | F | Y |  | Y |  | Y |
| M’Gonigle et al. 2015 | USA | CA | 2006 | 7 | 5 | 4.0 | N | 60 |  |  |  | 6143 |  |  |  | 97 |  |  |  | G, M, S | Y |  |  | Y | Y |
| Nayak et al. 2015 | UK |  | 2005 | 1 | 10 | 4 | V, P |  | 0.25 | 30 |  |  |  |  | 1774 |  |  |  | 55 | S | Y |  | Y | Y |  |
| Grass et al. 2016 | Germany |  | 2014 | 1 | 14 | 4 | N | 60 |  |  |  | 1015 |  |  |  | 42 |  |  |  | F, G, S | Y | Y |  | Y | Y |
| Zou et al. 2017 | China |  | 2015 | 1 | 18 | 5 | P |  | 10 |  |  |  | 3900 |  |  |  | 38 |  |  | . |  | Y | Y | Y | Y |
| Kremen et al. 2018 | USA | CA | 2006 | 9 | 15 | 3.5 | N | 60 |  |  |  | 7179 |  |  |  | 101 |  |  |  | M, G, S | Y |  |  | Y | Y |
| Pfiffner et al. 2018 | Switzerland |  | 2014 | 1 | 6 | 2 | N, P | 15 | 6.5 |  |  |  |  |  | 3973 |  |  |  | 91 | S |  | Y |  | Y | Y |
| Klein et al. 2012 | USA | CA | 2008 | 1 | 23 | 3 | V |  |  | 27 |  |  |  | 2794 |  |  |  | 20 |  | S | Y |  |  | Y | Y |
| Rollin et al. 2013 | France |  | 2010 | 3 | 812 | 1 | N | 15 |  |  |  | 29720 |  |  |  | 22 |  |  |  | G |  | Y |  | Y |  |
| Benjamin et al. 2014 | USA | NJ | 2010 | 3 | 16 | 3 | N | 60 |  |  |  | 1088 |  |  |  | 7 |  |  |  | G, M | Y | Y | Y |  |  |
|  | USA | NJ | 2010 | 3 | 16 | 3 | V |  |  | 60 |  |  |  | 1217 |  |  |  | 7 |  | G, M | Y | Y | Y |  |  |
| Cariveau et al. 2013 | USA | NJ | 2010 | 2 | 16 | 3 | V, N |  |  | 20 |  |  |  |  | 1017 |  |  |  | 4 | M, S |  | Y | Y |  |  |
|  | USA | NJ | 2010 | 2 | 16 | 2 | V, N | 60 |  |  |  |  |  |  | 2200 |  |  |  | 5 | M, S |  | Y | Y |  |  |
|  | USA | CA | 2010 | 2 | 12 | 3 | V, N | 68 |  | 40 |  |  |  |  | 1917 |  |  |  | 7 | M, S |  | Y | Y |  |  |
| Le Féon et al. 2013 | France |  | 2007 | 1 | 50 | 3 | P |  | 1.00 |  |  |  | 600 |  |  |  | 53 |  |  | S |  | Y |  |  |  |
| Bailey et al. 2014 | France |  | 2010 | 2 | 14 | 1 | P |  | 15.00 |  |  |  | 4594 |  |  |  | 83 |  |  | G, S | Y | Y |  |  |  |
| Hopfenmüller et al. 2014 | Germany |  | 2010 | 1 | 23 | 5 | N | 45 |  |  |  | 3469 |  |  |  | 189 |  |  |  | S |  | Y |  |  | Y |
| Riedinger et al. 2015 | Germany |  | 2011 | 2 | 16 | 2 | V |  |  | 15 |  |  |  | 426 |  |  |  | 46 |  | S | Y |  |  | Y |  |
| Connelly et al. 2015 | USA | NY | 2012 | 2 | 14 | 2 | P |  | 3.00 |  |  |  | 1075 |  |  |  | 65 |  |  | S |  | Y | Y |  |  |
| Scheper et al. 2015 | Germany, Sweden, Netherlands, UK |  | 2011 | 3 | 64 | 2 | V |  |  | 30 |  |  |  | 7173 |  |  |  | 125 |  | . | Y | Y |  | Y |  |
| Holland et al. 2015 | England |  | 2008 | 3 | 24 | 2 | V |  |  | . |  |  |  | 9 |  |  |  |  |  | M, S |  | Y |  | Y | Y |
| Williams et al. 2015 | USA | FL | 2010 | 2 | 9 | 8 | V, N | 20 |  | 20 |  |  |  |  | 7752 |  |  |  | 60 | S | Y |  |  | Y | Y |
|  | USA | MI | 2010 | 2 | 9 | 3 | V, N | 20 |  | 20 |  |  |  |  | 874 |  |  |  | 52 | S | Y |  |  | Y | Y |
|  | USA | CA | 2010 | 2 | 9 | 6 | V, N | 20 |  | 20 |  |  |  |  | 14750 |  |  |  | 80 | S | Y |  |  | Y | Y |
| Kremen and M’Gonigle 2015 | USA | CA | 2006 | 7 | 15 | 3 | N | 60 |  |  |  | 6145 |  |  |  | 97 |  |  |  | M, S | Y |  |  |  | Y |
| Mallinger et al. 2016 | USA | WI | 2010 | 3 | 30 | 3 | P |  | 7.00 |  |  |  | 8340 |  |  |  | 85 |  |  | S |  | Y |  | Y |  |
| Siregar et al. 2016 | Sumatra |  | 2012 | 1 | 3 | 3 | N | 388 |  |  |  | 31 |  |  |  |  |  |  |  | G, S |  | Y |  |  |  |
| Blitzer et al. 2016 | USA | NY | 2013 | 1 | 17 | 2 | N | 30 |  |  |  | 1579 |  |  |  | 53 |  |  |  | S |  | Y | Y | Y | Y |
| Harrison et al. 2019 | USA | NJ, NY, PA | 2013 | 3 | 36 | 4 | P |  | 1.00 |  |  |  | 12653 |  |  |  | 226 |  |  | S |  | Y |  |  | Y |
| Gervais et al. 2017 | Canada |  | 2013 | 2 | 12 | 4 | N, P | 20 | 2.00 |  |  |  |  |  | 1814 |  |  |  | 90 | S | Y | Y |  |  |  |
| Sutter et al. 2017 | Switzerland |  | 2013 | 2 | 8 | 2 | V |  |  | 20 |  |  |  | 3507 |  |  |  | 69 |  | S |  | Y |  | Y | Y |
| Bukovinszky et al. 2017 | Netherlands |  | 2012 | 1 | 20 | 2 | P |  | 2.00 |  |  |  | 532 |  |  |  | 44 |  |  | . |  | Y | Y | Y | Y |
| Nicholson et al. 2017 | USA | VT | 2013 | 3 | 15 | 3 | N | 10 |  |  |  | 1831 |  |  |  | 86 |  |  |  | M, S | Y | Y |  | Y |  |
|  | USA | VT | 2013 | 3 | 15 | 3 | V |  |  | 40 |  |  |  |  | 4706 |  |  | 89 |  | M, S | Y | Y |  | Y |  |
| Eeraerts et al. 2017 | Belgium |  | 2015 | 1 | 7 | 1 | N | 180 |  |  |  | 349 |  |  |  | 12 |  |  |  | S |  | Y | Y |  | Y |
| Morrison et al. 2017 | Spain |  | 2014 | 3 | 27 | 4 | P |  | 0.29 |  |  |  | 3489 |  |  |  | 26 | . |  | GG |  | Y |  | Y |  |
|  | Spain |  | 2014 | 3 | 27 | 4 | V |  |  | 25 |  |  |  | 524 |  |  |  | . |  | G |  | Y |  | Y |  |
| Warzecha et al. 2018 | Germany |  | 2013 | 2 | 28 | 5.5 | N | 15 |  |  |  | 1134 |  |  |  | 46 |  |  |  | S | Y |  |  | Y |  |
| Martins et al. 2018 | Canada |  | 2012 | 1 | 58 | 1 | N | 40 |  |  |  | 2348 |  |  |  | 8 |  |  |  | G, S |  | Y |  | Y | Y |
|  | Canada |  | 2012 | 1 | 58 | 1 | N | 40 |  |  |  | 781 |  |  |  | 6 |  |  |  | G, S |  | Y |  | Y | Y |
|  | Canada |  | 2012 | 1 | 58 | 1 | N | 40 |  |  |  | 5147 |  |  |  | 16 |  |  |  | G, S |  | Y |  | Y | Y |
| Macdonald et al. 2018 | New Zealand |  | 2015 | 1 | 20 | 7.0 | V |  |  | 10 |  |  |  | 365 |  |  |  | 4 |  | G, S |  | Y |  |  | Y |
| Tangtorwongsakul et al. 2018 | Thailand |  | 2015 | 1 | 24 | 1 | P |  | 0.33 |  |  |  | 3762 |  |  |  | 9 |  |  | G, S |  | Y |  | Y | Y |
|  | Thailand |  | 2015 | 1 | 24 | 1 | V |  |  | 120 |  |  |  | 3842 |  |  |  | 26 |  | G, S |  | Y |  | Y | Y |
| Tucker and Rehan 2018 | USA | NH | 2014 | 3 | 9 | 14 | N, P | 36 | 0.33 |  |  |  |  |  | 9105 |  |  |  | 205 | S |  | Y |  |  | Y |
| Ouvrard et al. 2018 | Belgium |  | 2014 | 2 | 4 | 6 | V |  |  | 30 |  |  |  | 1813 |  |  |  | 13 |  | S | Y |  |  | Y |  |
| Hipólito et al. 2018 | Brazil |  | 2013 | 2 | 34 | 2 | N | 20 |  |  |  | 115 |  |  |  | 19 |  |  |  | G, S |  | Y | Y |  |  |
| Hass et al. 2018 | France |  | 2013 | 1 | 30 | 2 | P |  | 4.00 |  |  |  | 2372 |  |  |  | 65 |  |  | M, S |  | Y | Y | Y |  |
|  | Germany |  | 2013 | 1 | 32 | 2 | P |  | 4.00 |  |  |  | 3354 |  |  |  | 85 |  |  | M, S |  | Y | Y | Y |  |
|  | Spain |  | 2013 | 1 | 20 | 2 | P |  | 4.00 |  |  |  | 3042 |  |  |  | 110 |  |  | M, S |  | Y | Y | Y |  |
|  | UK |  | 2013 | 1 | 12 | 2 | P |  | 4.00 |  |  |  | 545 |  |  |  | 9 |  |  | M, S |  | Y | Y | Y |  |
| Grab et al. 2018 | USA | NY | 2013 | 3 | 12 | 4 | V |  |  | 10 |  |  |  | 5684 |  |  |  | 99 |  | S | Y | Y | Y | Y | Y |
| Wood et al. 2018 | USA | MI | 2015 | 3 | 10 | 4 | N | 50 |  |  |  | 1546 |  |  |  | 109 |  |  |  | G, S | Y |  | Y | Y |  |
|  | USA | MI | 2015 | 3 | 10 | 3 | N | 50 |  |  |  | 840 |  |  |  | 79 |  |  |  | G, S | Y |  | Y | Y |  |
| Bartual et al. 2018 | Italy |  | 2015 | 2 | 25 | 1 | V |  |  | 20 |  |  |  | 2449 |  |  |  | 14 |  | M, S |  | Y | Y |  |  |
| Kremen et al. 2018 | USA | CA | 2006 | 9 | 15 | 4.0 | N | 60 |  |  |  | 7179 |  |  |  | 101 |  |  |  | M, S | Y |  |  | Y | Y |
| Breland et al. 2018 | USA | SC | 2016 | 1 | 126 | 3 | N, P | 20 | 1.00 |  |  |  |  |  | 1026 |  |  |  | 88 | S | Y |  | Y | Y |  |
| Happe et al. 2018 | Germany |  | 2013 | 1 | 36 | 3 | P |  | 7.00 |  |  |  | 1915 |  |  |  | 81 |  |  | S |  |  |  | Y | Y |
| Beduschi et al. 2018 | Germany |  | 2011 | 2 | 10 | 2 | P |  | 3.00 |  |  |  | 5241 |  |  |  | 93 |  |  | . | Y | Y |  | Y |  |
| Kratschmer et al. 2018 | Austria |  | 2016 | 2 | 16 | 5 | N | 15 |  |  |  | 493 |  |  |  | 84 |  |  |  | S |  | Y |  |  | Y |
| Ganser et al. 2018 | Switzerland |  | 2017 | 2 | 19 | 3 | V, N |  |  |  | 20 |  |  |  | 744 |  |  |  | 27 | G, S | Y | Y | Y | Y | Y |
| Mallinger et al. 2019 | USA | ND | 2016 | 2 | 20 | 8.5 | V |  |  | 5 |  |  |  | 4417 |  |  |  | 30 |  | M | Y | Y |  | Y |  |
| Fijen et al. 2019 | Italy |  | 2016 | 1 | 36 | 4.65 | V |  |  | 30 |  |  |  | 11525 |  |  |  | 171 |  | S | Y | Y |  | Y | Y |
| Castle et al. 2019 | Germany |  | 2016 | 1 | 21 | 3 | V, N | 5 |  | 5 |  |  |  |  | 18 |  |  |  | 4 | M, S | Y | Y | Y | Y | Y |
| Karamaouna et al. 2019 | Greece |  | 2011 | 3 | 12 | 3 | V |  |  | 12 |  |  |  | 155 |  |  |  | 3 |  | G | Y |  |  | Y | Y |
| Sirami et al. 2019 | France, UK, Germany, Spain, Canada |  | 2011 | 5 | 1305 | 2 | P |  | 4 |  |  |  | 13326 |  |  |  | 343 |  |  | S | Y | Y |  |  | Y |
| Xie et al. 2019 | China |  | 2017 | 1 | 11 | 3 | V |  |  | 10 |  |  |  | 4385 |  |  | 10 |  |  | G, S | Y | Y | Y |  | Y |
| Toivonen et al. 2019 | Finland |  | 2017 | 1 | 34 | 3 | V |  |  | 20 |  |  |  | 4188 |  |  |  | 9 |  | M, S | Y | Y | Y |  | Y |
| Cunningham-Minnick et al. 2019 | USA | OH | 2014 | 1 | 8 | 6 | P |  | 1 |  |  |  | 1724 |  |  |  | 53 |  |  | S |  |  | Y |  | Y |
| Eeraerts et al. 2019 | Belgium |  | 2016 | 2 | 18 | 1 | N | 225 |  |  |  | 1192 |  |  |  | 22 |  |  |  | S | Y | Y | Y | Y |  |
| Kratschmer et al. 2019 | Spain |  | 2016 | 1 | 16 | 5 | N | 15 |  |  |  | 134 |  |  |  | 20 |  |  |  | S |  | Y |  | Y | Y |
|  | France |  | 2016 | 1 | 15 | 5 | N | 15 |  |  |  | 181 |  |  |  | 35 |  |  |  | S |  | Y |  | Y | Y |
|  | Austria |  | 2016 | 1 | 16 | 5 | N | 15 |  |  |  | 329 |  |  |  | 64 |  |  |  | S |  | Y |  | Y | Y |
|  | Romania |  | 2016 | 1 | 16 | 5 | N | 15 |  |  |  | 77 |  |  |  | 38 |  |  |  | S |  | Y |  | Y | Y |
| Krimmer et al. 2019 | Germany |  | 2016 | 1 | 27 | 2 | N | 30 |  |  |  | 5862 |  |  |  | 56 |  |  |  | S | Y | Y |  | Y |  |
| Riojas-López et al. 2019 | Mexico |  | 2016 | 2 | 13 | 1 | P |  | 1.25 |  |  |  | 3823 |  |  |  | 45 |  |  | G, S | Y | Y |  |  |  |
| Sanchez et al. 2020 | Spain |  | 2011 | 2 | 6 | 7 | P |  | 14 |  |  |  | 6077 |  |  |  | 58 |  |  | F, G, M, S | Y | Y |  | Y |  |
| Brown et al. 2020 | Australia |  | 2017 | 1 | 14 | 4 | V |  |  | 40 |  |  |  | 3287 |  |  |  | 8 |  | M |  | Y |  | Y |  |
| Du Clos et al. 2020 | USA | ME | 2014 | 2 | 56 | 3 | N, P | 30 | 1 |  |  |  |  |  | 2094 |  |  |  | 135 | S | Y | Y |  | Y | Y |
| Jovani et al. 2020 | Mexico |  | 2014 | 1 | 4 | 12 | N | 60 |  |  |  | 2115 |  |  |  | 79 |  |  |  | S |  | Y |  |  |  |
| MacInnis et al. 2020 | Canada |  | 2018 | 1 | 12 | 2 | N | 15 |  |  |  | 784 |  |  |  | 70 |  |  |  | S | Y | Y |  | Y |  |
| McKerchar et al. 2020 | UK |  | 2013 | 4 | 10 | 3 | N | 10 |  |  |  | 1002 |  |  |  | 42 |  |  |  | S | Y |  | Y |  | Y |
| Levenson and Tarpy 2023 | USA | NC | 2016 | 3 | 12 | 4 | N | 60 |  |  |  | 11896 |  |  |  | 117 |  |  |  | G, S | Y | Y |  | Y | Y |
|  | USA | NC | 2016 | 3 | 16 | 4 | P |  | 0.25 |  |  |  | 4142 |  |  |  | 55 |  |  | G, S | Y | Y |  |  |  |
| Levenson and Tarpy 2022 | USA | NC | 2017 | 2 | 12 | 4 | N | 30 |  |  |  | 1643 |  |  |  | 28 |  |  |  | G, S | Y | Y |  | Y | Y |
| Levenson et al. 2022 | USA | NC | 2019 | 1 | 24 | 2.75 | N | 20 |  |  |  | 1617 |  |  |  | 51 |  |  |  | G, S | Y | Y | Y | Y |  |
|  | USA | NC | 2019 | 1 | 24 | 2.75 | V |  |  | 40 |  |  |  | 5601 |  |  |  | 26 |  | G, S | Y | Y | Y | Y |  |

**Citations**

**Bailey, S., F. Requier, B. Nusillard, S. P. M. Roberts, S. G. Potts, and C. Bouget**. **2014**. Distance from forest edge affects bee pollinators in oilseed rape fields. Ecol Evol. 4: 370–380.

**Bartual, A. M., G. Bocci, S. Marini, and A. C. Moonen**. **2018**. Local and landscape factors affect sunflower pollination in a Mediterranean agroecosystem. PLoS One. 13.

**Beduschi, T., U. G. Kormann, T. Tscharntke, and C. Scherber**. **2018**. Spatial community turnover of pollinators is relaxed by semi-natural habitats, but not by mass-flowering crops in agricultural landscapes. Biol Conserv. 221: 59–66.

**Benjamin, F. E., J. R. Reilly, and R. Winfree**. **2014**. Pollinator body size mediates the scale at which land use drives crop pollination services. Journal of Applied Ecology. 51: 440–449.

**Blaauw, B. R., and R. Isaacs**. **2014**. Flower plantings increase wild bee abundance and the pollination services provided to a pollination-dependent crop. Journal of Applied Ecology. 51: 890–898.

**Blitzer, E. J., J. Gibbs, M. G. Park, and B. N. Danforth**. **2016**. Pollination services for apple are dependent on diverse wild bee communities. Agric Ecosyst Environ. 221: 1–7.

**Breland, S., N. E. Turley, J. Gibbs, R. Isaacs, and L. A. Brudvig**. **2018**. Restoration increases bee abundance and richness but not pollination in remnant and post-agricultural woodlands. Ecosphere. 9.

**Brown, J., P. S. Barton, and S. A. Cunningham**. **2020**. Flower visitation and land cover associations of above ground- and below ground-nesting native bees in an agricultural region of south-east Australia. Agric Ecosyst Environ. 295.

**Bukovinszky, T., J. Verheijen, S. Zwerver, E. Klop, J. C. Biesmeijer, F. L. Wäckers, H. H. T. Prins, and D. Kleijn**. **2017**. Exploring the relationships between landscape complexity, wild bee species richness and reproduction, and pollination services along a complexity gradient in the Netherlands. Biol Conserv. 214: 312–319.

**Cariveau, D. P., N. M. Williams, F. E. Benjamin, and R. Winfree**. **2013**. Response diversity to land use occurs but does not consistently stabilise ecosystem services provided by native pollinators. Ecol Lett. 16: 903–911.

**Castle, D., I. Grass, and C. Westphal**. **2019**. Fruit quantity and quality of strawberries benefit from enhanced pollinator abundance at hedgerows in agricultural landscapes. Agric Ecosyst Environ. 275: 14–22.

**Du Clos, B., F. A. Drummond, and C. S. Loftin**. **2020**. Noncrop Habitat Use by Wild Bees (Hymenoptera: Apoidea) in a Mixed-Use Agricultural Landscape. Environ Entomol. 49: 502–515.

**Connelly, H., K. Poveda, and G. Loeb**. **2015**. Landscape simplification decreases wild bee pollination services to strawberry. Agric Ecosyst Environ. 211: 51–56.

**Cunningham-Minnick, M. J., V. E. Peters, and T. O. Crist**. **2019**. Nesting habitat enhancement for wild bees within soybean fields increases crop production. Apidologie. 50: 833–844.

**Eeraerts, M., I. Meeus, S. Van Den Berge, and G. Smagghe**. **2017**. Landscapes with high intensive fruit cultivation reduce wild pollinator services to sweet cherry. Agric Ecosyst Environ. 239: 342–348.

**Eeraerts, M., G. Smagghe, and I. Meeus**. **2019**. Pollinator diversity, floral resources and semi-natural habitat, instead of honey bees and intensive agriculture, enhance pollination service to sweet cherry. Agric Ecosyst Environ. 284.

**Le Féon, V., F. Burel, R. Chifflet, M. Henry, A. R. Vaissière, B. E. Vaissière, and J. Baudry**. **2013**. Solitary bee abundance and species richness in dynamic agricultural landscapes. Agric Ecosyst Environ. 166: 94–101.

**Fijen, T. P. M., J. A. Scheper, B. Boekelo, I. Raemakers, and D. Kleijn**. **2019**. Effects of landscape complexity on pollinators are moderated by pollinators’ association with mass-flowering crops. Proceedings of the Royal Society B: Biological Sciences. 286: 20190387.

**Ganser, D., B. Mayr, M. Albrecht, and E. Knop**. **2018**. Wildflower strips enhance pollination in adjacent strawberry crops at the small scale. Ecol Evol. 8: 11775–11784.

**Gervais, A., V. Fournier, C. S. Sheffield, and M. Chagnon**. **2017**. Assessing wild bee biodiversity in cranberry agroenvironments: Influence of natural habitats. J Econ Entomol. 110: 1424–1432.

**Grab, H., K. Poveda, B. Danforth, and G. Loeb**. **2018**. Landscape context shifts the balance of costs and benefits from wildflower borders on multiple ecosystem services. Proceedings of the Royal Society B: Biological Sciences. 285.

**Grass, I., J. Albrecht, F. Jauker, T. Diekötter, D. Warzecha, V. Wolters, and N. Farwig**. **2016**. Much more than bees-Wildflower plantings support highly diverse flower-visitor communities from complex to structurally simple agricultural landscapes. Agric Ecosyst Environ. 225: 45–53.

**Hagen, M., and M. Kraemer**. **2010**. Agricultural surroundings support flower–visitor networks in an Afrotropical rain forest. Biol Conserv. 143: 1654–1663.

**Hannon, L. E., and T. D. Sisk**. **2009**. Hedgerows in an agri-natural landscape: Potential habitat value for native bees. Biol Conserv. 142: 2140–2154.

**Happe, A. K., F. Riesch, V. Rösch, R. Gallé, T. Tscharntke, and P. Batáry**. **2018**. Small-scale agricultural landscapes and organic management support wild bee communities of cereal field boundaries. Agric Ecosyst Environ. 254: 92–98.

**Harrison, T., J. Gibbs, and R. Winfree**. **2019**. Anthropogenic landscapes support fewer rare bee species. Landsc Ecol. 34: 967–978.

**Hass, A. L., U. G. Kormann, T. Tscharntke, Y. Clough, A. B. Baillod, C. Sirami, L. Fahrig, J. L. Martin, J. Baudry, C. Bertrand, J. Bosch, L. Brotons, F. Bure, R. Georges, D. Giralt, M. Marcos-García, A. Ricarte, G. Siriwardena, and P. Batáry**. **2018**. Landscape configurational heterogeneity by small-scale agriculture, not crop diversity, maintains pollinators and plant reproduction in western Europe. Proceedings of the Royal Society B: Biological Sciences. 285.

**Hipólito, J., D. Boscolo, and B. F. Viana**. **2018**. Landscape and crop management strategies to conserve pollination services and increase yields in tropical coffee farms. Agric Ecosyst Environ. 256: 218–225.

**Holland, J. M., B. M. Smith, J. Storkey, P. J. W. Lutman, and N. J. Aebischer**. **2015**. Managing habitats on English farmland for insect pollinator conservation. Biol Conserv. 182: 215–222.

**Hopfenmüller, S., I. Steffan-Dewenter, and A. Holzschuh**. **2014**. Trait-specific responses of wild bee communities to landscape composition, configuration and local factors. PLoS One. 9: 104439.

**Jha, S., and J. H. Vandermeer**. **2010**. Impacts of coffee agroforestry management on tropical bee communities. Biol Conserv. 143: 1423–1431.

**Jovani, R.-T., V. Rémy, P.-N. Patricia, G. Jaime, and S. Daniel**. **2020**. Seasonal abundance and diversity of native bees in a patchy agricultural landscape in Southern Mexico. Agric Ecosyst Environ. 106807.

**Karamaouna, F., V. Kati, N. Volakakis, K. Varikou, N. Garantonakis, L. Economou, A. Birouraki, E. Markellou, S. Liberopoulou, and M. Edwards**. **2019**. Ground cover management with mixtures of flowering plants to enhance insect pollinators and natural enemies of pests in olive groves. Agric Ecosyst Environ. 274: 76–89.

**Klein, A. M., C. Brittain, S. D. Hendrix, R. Thorp, N. Williams, and C. Kremen**. **2012**. Wild pollination services to California almond rely on semi-natural habitat. Journal of Applied Ecology. 49: 723–732.

**Klein, A.-M., I. Steffan-Dewenter, and T. Tscharntke**. **2003**. Fruit set of highland coffee increases with the diversity of pollinating bees. Proceedings of the Royal Society London, Series B. 270: 955–61.

**Kratschmer, S., B. Pachinger, M. Schwantzer, D. Paredes, M. Guernion, F. Burel, A. Nicolai, P. Strauss, T. Bauer, M. Kriechbaum, J. G. Zaller, and S. Winter**. **2018**. Tillage intensity or landscape features: What matters most for wild bee diversity in vineyards? Agric Ecosyst Environ. 266: 142–152.

**Kratschmer, S., B. Pachinger, M. Schwantzer, D. Paredes, G. Guzmán, J. A. Goméz, J. A. Entrenas, M. Guernion, F. Burel, A. Nicolai, A. Fertil, D. Popescu, L. Macavei, A. Hoble, C. Bunea, M. Kriechbaum, J. G. Zaller, and S. Winter**. **2019**. Response of wild bee diversity, abundance, and functional traits to vineyard inter-row management intensity and landscape diversity across Europe. Ecol Evol. 9: 4103–4115.

**Kremen, C., and L. K. M’Gonigle**. **2015**. Small-scale restoration in intensive agricultural landscapes supports more specialized and less mobile pollinator species. Journal of Applied Ecology. 52: 602–610.

**Kremen, C., L. K. M’Gonigle, and L. C. Ponisio**. **2018**. Pollinator community assembly tracks changes in floral resources as restored hedgerows mature in agricultural landscapes. Front Ecol Evol. 6: 170.

**Krimmer, E., E. A. Martin, J. Krauss, A. Holzschuh, and I. Steffan-Dewenter**. **2019**. Size, age and surrounding semi-natural habitats modulate the effectiveness of flower-rich agri-environment schemes to promote pollinator visitation in crop fields. Agric Ecosyst Environ. 284.

**Levenson, H. K., A. E. Sharp, and D. R. Tarpy**. **2022**. Evaluating the impact of increased pollinator habitat on bee visitation and yield metrics in soybean crops. Agric Ecosyst Environ. 331.

**Levenson, H. K., and D. R. Tarpy**. **2022**. Effects of planted pollinator habitat on pathogen prevalence and interspecific detection between bee species. Sci Rep. 12.

**Levenson, H. K., and D. R. Tarpy**. **2023**. Planted pollinator habitat in agroecosystems: How does the pollinator community respond? Front Ecol Evol. 11.

**Macdonald, K. J., D. Kelly, and J. M. Tylianakis**. **2018**. Do local landscape features affect wild pollinator abundance, diversity and community composition on Canterbury farms? N Z J Ecol. 42: 262–268.

**MacInnis, G., C. M. Buddle, and J. R. K. Forrest**. **2020**. Small wild bee abundance declines with distance into strawberry crops regardless of field margin habitat. Basic Appl Ecol. 44: 14–23.

**Mallinger, R. E., J. G. Franco, D. A. Prischmann-Voldseth, and J. R. Prasifka**. **2019**. Annual cover crops for managed and wild bees: Optimal plant mixtures depend on pollinator enhancement goals. Agric Ecosyst Environ. 273: 107–116.

**Mallinger, R. E., J. Gibbs, and C. Gratton**. **2016**. Diverse landscapes have a higher abundance and species richness of spring wild bees by providing complementary floral resources over bees’ foraging periods. Landsc Ecol. 31: 1523–1535.

**Martins, K. T., E. H. Albert, M. J. Lechowicz, and A. Gonzalez**. **2018**. Complementary crops and landscape features sustain wild bee communities. Ecological Applications. 28: 1093–1105.

**McKerchar, M., S. G. Potts, M. T. Fountain, M. P. D. Garratt, and D. B. Westbury**. **2020**. The potential for wildflower interventions to enhance natural enemies and pollinators in commercial apple orchards is limited by other management practices. Agric Ecosyst Environ. 301.

**M’Gonigle, L. K., L. C. Ponisio, K. Cutler, and C. Kremen**. **2015**. Habitat restoration promotes pollinator persistence and colonization in intensively managed agriculture. Ecological Applications. 25: 1557–1565.

**Morrison, J., J. Izquierdo, E. H. Plaza, and J. L. González-Andújar**. **2017**. The role of field margins in supporting wild bees in Mediterranean cereal agroecosystems: Which biotic and abiotic factors are important? Agric Ecosyst Environ. 247: 216–224.

**Nayak, G. K., S. P. M. Roberts, M. Garratt, T. D. Breeze, T. Tscheulin, J. Harrison-Cripps, I. N. Vogiatzakis, M. T. Stirpe, and S. G. Potts**. **2015**. Interactive effect of floral abundance and semi-natural habitats on pollinators in field beans (Vicia faba). Agric Ecosyst Environ. 199: 58–66.

**Nicholson, C. C., I. Koh, L. L. Richardson, A. Beauchemin, and T. H. Ricketts**. **2017**. Farm and landscape factors interact to affect the supply of pollination services. Agric Ecosyst Environ. 250: 113–122.

**Ouvrard, P., J. Transon, and A.-L. Jacquemart**. **2018**. Flower-strip agri-environment schemes provide diverse and valuable summer flower resources for pollinating insects. Biodivers Conserv. 27: 2193–2216.

**Pfiffner, L., M. Ostermaier, S. Stoeckli, and A. Müller**. **2018**. Wild bees respond complementarily to ‘high-quality’ perennial and annual habitats of organic farms in a complex landscape. J Insect Conserv. 22: 551–562.

**Riedinger, V., O. Mitesser, T. Hovestadt, I. Steffan-Dewenter, and A. Holzschuh**. **2015**. Annual dynamics of wild bee densities: attractiveness and productivity effects of oilseed rape. Ecology. 96: 1351–1360.

**Riojas-López, M. E., I. A. Díaz-Herrera, H. E. Fierros-López, and E. Mellink**. **2019**. The effect of adjacent habitat on native bee assemblages in a perennial low-input agroecosystem in a semiarid anthropized landscape. Agric Ecosyst Environ. 272: 199–205.

**Rollin, O., V. Bretagnolle, A. Decourtye, J. Aptel, N. Michel, B. E. Vaissière, and M. Henry**. **2013**. Differences of floral resource use between honey bees and wild bees in an intensive farming system. Agric Ecosyst Environ. 179: 78–86.

**Sanchez, J. A., A. Carrasco, M. La Spina, M. Pérez-Marcos, and F. J. Ortiz-Sánchez**. **2020**. How bees respond differently to field margins of shrubby and herbaceous plants in intensive agricultural crops of the mediterranean area. Insects. 11.

**Scheper, J., R. Bommarco, A. Holzschuh, S. G. Potts, V. Riedinger, S. P. M. Roberts, M. Rundlöf, H. G. Smith, I. Steffan-Dewenter, J. B. Wickens, V. J. Wickens, and D. Kleijn**. **2015**. Local and landscape-level floral resources explain effects of wildflower strips on wild bees across four European countries. Journal of Applied Ecology. 52: 1165–1175.

**Sirami, C., N. Gross, A. B. Baillod, C. Bertrand, R. Carrié, A. Hass, L. Henckel, P. Miguet, C. Vuillot, A. Alignier, J. Girard, P. Batáry, Y. Clough, C. Violle, D. Giralt, G. Bota, I. Badenhausser, G. Lefebvre, B. Gauffre, A. Vialatte, F. Calatayud, A. Gil-Tena, L. Tischendorf, S. Mitchell, K. Lindsay, R. Georges, S. Hilaire, J. Recasens, X. O. Solé-Senan, I. Robleño, J. Bosch, J. A. Barrientos, A. Ricarte, M. Á. Marcos-Garcia, J. Miñano, R. Mathevet, A. Gibon, J. Baudry, G. Balent, B. Poulin, F. Burel, T. Tscharntke, V. Bretagnolle, G. Siriwardena, A. Ouin, L. Brotons, J. L. Martin, and L. Fahrig**. **2019**. Increasing crop heterogeneity enhances multitrophic diversity across agricultural regions. Proc Natl Acad Sci U S A. 116: 16442–16447.

**Siregar, E. H., T. Atmowidi, and S. Kahono**. **2016**. Diversity and Abundance of Insect Pollinators in Different Agricultural Lands in Jambi, Sumatera. Hayati. 23: 13–17.

**Sutter, L., P. Jeanneret, A. M. Bartual, G. Bocci, and M. Albrecht**. **2017**. Enhancing plant diversity in agricultural landscapes promotes both rare bees and dominant crop-pollinating bees through complementary increase in key floral resources. Journal of Applied Ecology. 54: 1856–1864.

**Tangtorwongsakul, P., N. Warrit, and G. A. Gale**. **2018**. Effects of landscape cover and local habitat characteristics on visiting bees in tropical orchards. Agric For Entomol. 20: 28–40.

**Toivonen, M., I. Herzon, H. Rajanen, J. Toikkanen, and M. Kuussaari**. **2019**. Late flowering time enhances insect pollination of turnip rape. Journal of Applied Ecology. 56: 1164–1175.

**Tucker, E. M., and S. M. Rehan**. **2018**. Farming for bees: annual variation in pollinator populations across agricultural landscapes. Agric For Entomol. 20: 541–548.

**Warzecha, D., T. Diekötter, V. Wolters, and F. Jauker**. **2018**. Attractiveness of wildflower mixtures for wild bees and hoverflies depends on some key plant species. Insect Conserv Divers. 11: 32–41.

**Williams, N. M., K. L. Ward, N. Pope, R. Isaacs, J. Wilson, E. A. May, J. Ellis, J. Daniels, A. Pence, K. Ullmann, and J. Peters**. **2015**. Native wildflower plantings support wild bee abundance and diversity in agricultural landscapes across the United States. Ecological Applications. 25: 2119–2131.

**Wood, T. J., J. Gibbs, N. Rothwell, J. K. Wilson, L. Gut, J. Brokaw, and R. Isaacs**. **2018**. Limited phenological and dietary overlap between bee communities in spring flowering crops and herbaceous enhancements. Ecological Applications. 28: 1924–1934.

**Xie, Z., J. Wang, D. Pan, and J. An**. **2019**. Landscape-modified concentration effect and waylaying effect of bees and their consequences on pollination of mass-flowering plants in agricultural ecosystems. Agric Ecosyst Environ. 280: 24–34.

**Zou, Y., F. J. J. A. Bianchi, F. Jauker, H. Xiao, J. Chen, J. Cresswell, S. Luo, J. Huang, X. Deng, L. Hou, and W. van der Werf**. **2017**. Landscape effects on pollinator communities and pollination services in small-holder agroecosystems. Agric Ecosyst Environ. 246: 109–116.
