# Supplementary material for: Effects of study design parameters on estimates of bee abundance and richness in agroecosystems: a meta-analysis
Source: Ann Entomol Soc Am. 2024 Jan 19;117(2):92–106. doi: 10.1093/aesa/saae001 (PMC10933562; doi:10.1093/aesa/saae001)
Supplement: saae001_suppl_Supplementary_Table_S3 [file saae001_suppl_supplementary_table_s3.docx]

Effects of study design parameters on estimates of bee abundance and richness in agroecosystems: a meta-analysis

Hannah K. Levenson^1^*, Bradley N. Metz^2^, David R. Tarpy^2^

1. Department of Entomology and Plant Pathology, North Carolina State University, NC, USA

2. Department of Applied Ecology, North Carolina State University, NC, USA

*Corresponding Author: 2301 Gardner Hall, 100 Derieux Place, North Carolina State University, Raleigh NC, 27695; 919.434.7882; [hklevens@ncsu.edu](mailto:hklevens@ncsu.edu)

**Supp. Table S3.** Lists the publications not included in our final corpus, at what stage in the screening process the publication was excluded, and the reason for exclusion.

| **Paper Number** | **Screening Stage** | **Reason For Exclusion** |
| --- | --- | --- |
| Feltham et al. 2015 | 3 | Variable of interest not reported or easily accessible |
| Wood et al. 2015 | 3 | Variable of interest not reported or easily accessible |
| Greenleaf and Kremen 2006 | 3 | Variable of interest not reported or easily accessible |
| Shuler et al. 2005 | 3 | Variable of interest not reported or easily accessible |
| Winfree et al. 2008 | 3 | Sampling methods not standardized |
| Bartomeus et al. 2014 | 3 | Variable of interest not reported or easily accessible |
| Brandt et al. 2017 | 3 | Variable of interest not reported or easily accessible |
| Fründ et al. 2010 | 3 | Variable of interest not reported or easily accessible |
| Korpela et al. 2013 | 2 | Focus limited to group(s), not entire community |
| Garibaldi et al. 2011 | 2 | Not primary, empirical research |
| Carvalheiro et al. 2012 | 3 | Variable of interest not reported or easily accessible |
| Holzschuh et al. 2012 | 3 | Variable of interest not reported or easily accessible |
| Blitzer et al. 2012 | 2 | Not primary, empirical research |
| Peters and Carroll 2012 | 3 | Variable of interest not reported or easily accessible |
| Kennedy et al. 2013 | 2 | Not primary, empirical research |
| Morandin and Kremen 2013 | 3 | Variable of interest not reported or easily accessible |
| Ferreira et al. 2013 | 2 | Not primary, empirical research |
| Garibaldi et al. 2014 | 2 | Not primary, empirical research |
| Blaauw and Isaacs 2014 | 3 | Variable of interest not reported or easily accessible |
| Cusser et al. 2015 | 3 | Variable of interest not reported or easily accessible |
| Jönsson et al. 2015 | 3 | Sampling methods not standardized |
| Senapathi et al. 2015 | 3 | Sampling methods not standardized |
| Pywell et al. 2015 | 3 | Variable of interest not reported or easily accessible |
| Hardman et al. 2016 | 3 | Variable of interest not reported or easily accessible |
| Dainese et al. 2018 | 3 | Variable of interest not reported or easily accessible |
| Garibaldi et al. 2016 | 2 | Not primary, empirical research |
| Lichtenberg et al. 2017 | 2 | Not primary, empirical research |
| Campbell et al. 2017 | 3 | Variable of interest not reported or easily accessible |
| Cole et al. 2017 | 2 | Focus limited to group(s), not entire community |
| Uyttenbroeck et al. 2017 | 3 | Variable of interest not reported or easily accessible |
| Venturini et al. 2017 | 2 | Not primary, empirical research |
| Wood et al. 2017 | 3 | Sampling methods not standardized |
| Papanikolaou, Kühn, et al. 2017 | 3 | Sampling methods not standardized |
| Horth and Campbell 2018 | 2 | Focus limited to group(s), not entire community |
| Garratt et al. 2017 | 3 | Variable of interest not reported or easily accessible |
| Papanikolaou, Kuhn, et al. 2017 | 3 | Sampling methods not standardized |
| Ouvrard and Jacquemart 2018 | 3 | Variable of interest not reported or easily accessible |
| Rundlöf et al. 2018 | 3 | Variable of interest not reported or easily accessible |
| Collado et al. 2019 | 3 | Variable of interest not reported or easily accessible |
| Buhk et al. 2018 | 3 | Variable of interest not reported or easily accessible |
| Amy et al. 2018 | 3 | Variable of interest not reported or easily accessible |
| Grab, Brokaw, Anderson, Gedlinske, Gibbs, Wilson, Loeb, Rufus Isaacs, et al. 2019 | 2 | Focus limited to group(s), not entire community |
| Tonietto and Larkin 2018 | 2 | Not primary, empirical research |
| Wietzke et al. 2018 | 2 | Did not measure beyond crop |
| Fijen et al. 2018 | 3 | Variable of interest not reported or easily accessible |
| Garratt et al. 2018 | 3 | Variable of interest not reported or easily accessible |
| Filipiak 2019 | 2 | Focus limited to group(s), not entire community |
| Grass et al. 2018 | 3 | Variable of interest not reported or easily accessible |
| Timberlake et al. 2019 | 2 | Focus limited to group(s), not entire community |
| Redhead et al. 2018 | 2 | Not primary, empirical research |
| Mach and Potter 2018 | 2 | Not conducted in agricultural settings |
| Coutinho et al. 2018 | 2 | Not primary, empirical research |
| Indah Setyawati et al. 2014 | 3 | Variable of interest not reported or easily accessible |
| Dainese et al. 2019 | 2 | Not primary, empirical research |
| Gilpin et al. 2019 | 3 | Focus limited to group(s), not entire community |
| Marja et al. 2019 | 2 | Not primary, empirical research |
| Vanderplanck et al. 2019 | 3 | Focus limited to group(s), not entire community |
| Nicholson et al. 2019 | 2 | Not primary, empirical research |
| Brown and Cunningham 2019 | 2 | Not primary, empirical research |
| Burdine and McCluney 2019 | 2 | Not conducted in agricultural settings |
| Cusser et al. 2019 | 3 | Focus limited to group(s), not entire community |
| Woodcock et al. 2019 | 2 | Not primary, empirical research |
| Mkenda et al. 2019 | 2 | Not primary, empirical research |
| Rollings and Goulson 2019 | 2 | Focus limited to group(s), not entire community |
| Martin et al. 2019 | 2 | Not primary, empirical research |
| Huang and D’Odorico 2020 | 2 | Not primary, empirical research |
| Aguilera et al. 2020 | 2 | Not primary, empirical research |
| Gardner et al. 2021 | 2 | Not primary, empirical research |
| Zamorano et al. 2020 | 2 | Not primary, empirical research |
| Bennett et al. 2020 | 2 | Not primary, empirical research |
| Kline and Joshi 2020 | 2 | Not primary, empirical research |
| Bogusch et al. 2020 | 2 | Not conducted in agricultural settings |
| Rader et al. 2020 | 2 | Not primary, empirical research |
| Porto et al. 2020 | 2 | Not primary, empirical research |
| Doré et al. 2021 | 2 | Not primary, empirical research |
| Schwarz et al. 2020 | 2 | Not primary, empirical research |
| Albrecht et al. 2020 | 2 | Not primary, empirical research |
| Bartomeus et al. 2018 | 3 | Variable of interest not reported or easily accessible |
| Grab, Branstetter, et al. 2019 | 3 | Variable of interest not reported or easily accessible |
| Catarino et al. 2019 | 3 | Variable of interest not reported or easily accessible |
| Heller et al. 2019 | 3 | Sampling method not considered in our analysis |
| Arathi et al. 2019 | 3 | Sampling method not considered in our analysis |
| Sritongchuay et al. 2019 | 3 | Variable of interest not reported or easily accessible |
| Grab, Brokaw, Anderson, Gedlinske, Gibbs, Wilson, Loeb, Isaacs, et al. 2019 | 3 | Variable of interest not reported or easily accessible |
| Lázaro and Alomar 2019 | 3 | Variable of interest not reported or easily accessible |
| Medeiros et al. 2019 | 3 | Sampling method not considered in our analysis |
| Cely-Santos and Philpott 2019 | 3 | Variable of interest not reported or easily accessible |
| Saunders and Rader 2019 | 3 | Variable of interest not reported or easily accessible |
| Knapp et al. 2019 | 2 | Focus limited to group(s), not entire community |
| Rollin et al. 2019 | 2 | Not primary, empirical research |
| Paterson et al. 2019 | 3 | Variable of interest not reported or easily accessible |
| Reverté et al. 2019 | 3 | Variable of interest not reported or easily accessible |
| Nichols et al. 2019 | 3 | Variable of interest not reported or easily accessible |
| Bartual et al. 2019 | 3 | Variable of interest not reported or easily accessible |
| Nicholson and Ricketts 2019 | 2 | Not primary, empirical research |
| Campbell et al. 2019 | 3 | Variable of interest not reported or easily accessible |
| Kay et al. 2020 | 2 | Not primary, empirical research |
| Griffiths-Lee et al. 2020 | 3 | Variable of interest not reported or easily accessible |
| Reilly et al. 2020 | 3 | Variable of interest not reported or easily accessible |
| Uzman et al. 2020 | 2 | Focus limited to group(s), not entire community |
| Ropars et al. 2020 | 3 | Variable of interest not reported or easily accessible |
| Shaw et al. 2020 | 3 | Sampling methods not standardized |
| Bartholomée et al. 2019 | 3 | Variable of interest not reported or easily accessible |
| Chatterjee et al. 2020 | 2 | Not primary, empirical research |
| Liu et al. 2020 | 3 | Variable of interest not reported or easily accessible |
| Main et al. 2020 | 3 | Sampling method not considered in our analysis |
| Burkle et al. 2020 | 3 | Variable of interest not reported or easily accessible |
| Varah et al. 2020 | 2 | Focus limited to group(s), not entire community |
| McCallum et al. 2021 | 3 | Variable of interest not reported or easily accessible |
| Kordbacheh et al. 2020 | 3 | Sampling method not considered in our analysis |

**Citations**

**Aguilera, G., T. Roslin, K. Miller, G. Tamburini, K. Birkhofer, B. Caballero-Lopez, S. A. M. Lindström, E. Öckinger, M. Rundlöf, A. Rusch, H. G. Smith, and R. Bommarco**. **2020**. Crop diversity benefits carabid and pollinator communities in landscapes with semi-natural habitats. Journal of Applied Ecology. 57: 2170–2179.

**Albrecht, M., D. Kleijn, N. M. Williams, M. Tschumi, B. R. Blaauw, R. Bommarco, A. J. Campbell, M. Dainese, F. A. Drummond, M. H. Entling, D. Ganser, G. Arjen De Groot, D. Goulson, H. Grab, H. Hamilton, F. Herzog, R. Isaacs, K. Jacot, P. Jeanneret, M. Jonsson, E. Knop, C. Kremen, D. A. Landis, G. M. Loeb, L. Marini, M. Mckerchar, L. Morandin, S. C. Pfister, A. Sciligo, C. Thies, T. Tscharntke, E. Venturini, E. Veromann, I. M. G. Vollhardt, F. W€, K. Ward, A. Wilby, M. Woltz, S. Wratten, and L. Sutter**. **2020**. The effectiveness of flower strips and hedgerows on pest control, pollination services and crop yield: a quantitative synthesis. Ecol Lett. 23: 1488–1498.

**Amy, C., G. Noël, S. Hatt, R. Uyttenbroeck, F. Van De Meutter, D. Genoud, and F. Francis**. **2018**. Flower strips in wheat intercropping system: Effect on pollinator abundance and diversity in Belgium. Insects. 9: 114.

**Arathi, H. S., M. W. Vandever, and B. S. Cade**. **2019**. Diversity and abundance of wild bees in an agriculturally dominated landscape of eastern Colorado. J Insect Conserv. 23: 187–197.

**Bartholomée, O., A. Aullo, J. Becquet, C. Vannier, and S. Lavorel**. **2019**. Pollinator presence in orchards depends on landscape-scale habitats more than in-field flower resources. Agric Ecosyst Environ. 293.

**Bartomeus, I., D. P. Cariveau, T. Harrison, and R. Winfree**. **2018**. On the inconsistency of pollinator species traits for predicting either response to land-use change or functional contribution. Oikos. 127: 306–315.

**Bartomeus, I., S. G. Potts, I. Steffan-Dewenter, B. E. Vaissière, M. Woyciechowski, K. M. Krewenka, T. Tscheulin, S. P. M. Roberts, H. Szentgyörgyi, C. Westphal, and R. Bommarco**. **2014**. Contribution of insect pollinators to crop yield and quality varies with agricultural intensification. PeerJ. 2014: e328.

**Bartual, A. M., L. Sutter, G. Bocci, A. C. Moonen, J. Cresswell, M. Entling, B. Giffard, K. Jacot, P. Jeanneret, J. Holland, S. Pfister, O. Pintér, E. Veromann, K. Winkler, and M. Albrecht**. **2019**. The potential of different semi-natural habitats to sustain pollinators and natural enemies in European agricultural landscapes. Agric Ecosyst Environ. 279: 43–52.

**Bennett, J. M., J. A. Steets, J. H. Burns, L. A. Burkle, J. C. Vamosi, M. Wolowski, G. Arceo-Gómez, M. Burd, W. Durka, A. G. Ellis, L. Freitas, J. Li, J. G. Rodger, V. Ştefan, J. Xia, T. M. Knight, and T. L. Ashman**. **2020**. Land use and pollinator dependency drives global patterns of pollen limitation in the Anthropocene. Nat Commun. 11: 20.

**Blaauw, B. R., and R. Isaacs**. **2014**. Larger patches of diverse floral resources increase insect pollinator density, diversity, and their pollination of native wildflowers. Basic Appl Ecol. 15: 701–711.

**Blitzer, E. J., C. F. Dormann, A. Holzschuh, A. M. Klein, T. A. Rand, and T. Tscharntke**. **2012**. Spillover of functionally important organisms between managed and natural habitats. Agric Ecosyst Environ. 146: 34–43.

**Bogusch, P., L. Hlaváčková, N. Rodriguez Gasol, and P. Heneberg**. **2020**. Near-natural habitats near almond orchards with presence of empty gastropod shells are important for solitary shell-nesting bees and wasps. Agric Ecosyst Environ. 299: 106949.

**Brandt, K., M. Glemnitz, and B. Schröder**. **2017**. The impact of crop parameters and surrounding habitats on different pollinator group abundance on agricultural fields. Agric Ecosyst Environ. 243: 55–66.

**Brown, J., and S. A. Cunningham**. **2019**. Global-scale drivers of crop visitor diversity and the historical development of agriculture. Proceedings of the Royal Society B: Biological Sciences. 286.

**Buhk, C., R. Oppermann, A. Schanowski, R. Bleil, J. Lüdemann, and C. Maus**. **2018**. Flower strip networks offer promising long term effects on pollinator species richness in intensively cultivated agricultural areas. BMC Ecol. 18.

**Burdine, J. D., and K. E. McCluney**. **2019**. Interactive effects of urbanization and local habitat characteristics influence bee communities and flower visitation rates. Oecologia. 190: 715–723.

**Burkle, L. A., C. M. Delphia, K. M. O’neill, and L. A. Burkle**. **2020**. Redundancy in wildflower strip species helps support spatiotemporal variation in wild bee communities on diversified farms. Basic Appl Ecol. 44: 1–13.

**Campbell, A. J., A. Wilby, P. Sutton, and F. L. Wäckers**. **2017**. Do sown flower strips boost wild pollinator abundance and pollination services in a spring-flowering crop? A case study from UK cider apple orchards. Agric Ecosyst Environ. 239: 20–29.

**Campbell, J. W., C. B. Kimmel, S. M. Grodsky, C. Smithers, J. C. Daniels, and J. D. Ellis**. **2019**. Wildflower plantings harbor increased arthropod richness and abundance within agricultural areas in Florida (USA). Ecosphere. 10.

**Carvalheiro, L. G., C. L. Seymour, S. W. Nicolson, and R. Veldtman**. **2012**. Creating patches of native flowers facilitates crop pollination in large agricultural fields: Mango as a case study. Journal of Applied Ecology. 49: 1373–1383.

**Catarino, R., V. Bretagnolle, T. Perrot, F. Vialloux, and S. Gaba**. **2019**. Bee pollination outperforms pesticides for oilseed crop production and profitability. Proceedings of the Royal Society B: Biological Sciences. 286.

**Cely-Santos, M., and S. M. Philpott**. **2019**. Local and landscape habitat influences on bee diversity in agricultural landscapes in Anolaima, Colombia. J Insect Conserv. 23: 133–146.

**Chatterjee, A., S. Chatterjee, B. Smith, J. E. Cresswell, and P. Basu**. **2020**. Predicted thresholds for natural vegetation cover to safeguard pollinator services in agricultural landscapes. Agric Ecosyst Environ. 290.

**Cole, L. J., S. Brocklehurst, D. Robertson, W. Harrison, and D. I. McCracken**. **2017**. Exploring the interactions between resource availability and the utilisation of semi-natural habitats by insect pollinators in an intensive agricultural landscape. Agric Ecosyst Environ. 246: 157–167.

**Collado, M., D. Sol, and I. Bartomeus**. **2019**. Bees use anthropogenic habitats despite strong natural habitat preferences. Divers Distrib. 25: 924–935.

**Coutinho, J. G. da E., L. A. Garibaldi, and B. F. Viana**. **2018**. The influence of local and landscape scale on single response traits in bees: A meta-analysis. Agric Ecosyst Environ. 256: 61–73.

**Cusser, S., J. L. Neff, and S. Jha**. **2015**. Land use change and pollinator extinction debt in exurban landscapes. Insect Conserv Divers. 8: 562–572.

**Cusser, S., J. L. Neff, and S. Jha**. **2019**. Landscape context differentially drives diet breadth for two key pollinator species. Oecologia. 191: 873–886.

**Dainese, M., E. A. Martin, M. A. Aizen, M. Albrecht, I. Bartomeus, R. Bommarco, L. G. Carvalheiro, R. Chaplin-Kramer, V. Gagic, L. A. Garibaldi, J. Ghazoul, H. Grab, M. Jonsson, D. S. Karp, C. M. Kennedy, D. Kleijn, C. Kremen, D. A. Landis, D. K. Letourneau, A. Diego, M. Bezerra, F. J. J. A. Bianchi, V. Boreux, V. Bretagnolle, B. Caballero-Lopez, P. Cavigliasso, A. Ćetković, N. P. Chacoff, A. Classen, S. Cusser, F. D. Da Silva E Silva, G. Arjen De Groot, J. H. Dudenhöffer, J. Ekroos, T. Fijen, P. Franck, B. M. Freitas, M. P. D. Garratt, C. Gratton, J. Hipólito, A. Holzschuh, L. Hunt, A. L. Iverson, S. Jha, T. Keasar, T. N. Kim, M. Kishinevsky, B. K. Klatt, A.-M. Klein, K. M. Krewenka, S. Krishnan, A. E. Larsen, C. Lavigne, H. Liere, B. Maas, R. E. Mallinger, E. M. Pachon, A. Martínez-Salinas, T. D. Meehan, M. G. E. Mitchell, G. A. R. Molina, M. Nesper, L. Nilsson, M. E. O’rourke, M. K. Peters, M. Plećaš, S. G. Potts, D. De, L. Ramos, J. A. Rosenheim, M. Rundlöf, A. Rusch, A. Sáez, J. Scheper, M. Schleuning, J. M. Schmack, A. R. Sciligo, C. Seymour, D. A. Stanley, R. Stewart, J. C. Stout, L. Sutter, M. B. Takada, H. Taki, G. Tamburini, M. Tschumi, B. F. Viana, C. Westphal, B. K. Willcox, S. D. Wratten, A. Yoshioka, C. Zaragoza-Trello, W. Zhang, Y. Zou, and I. Steffan-Dewenter**. **2019**. A global synthesis reveals biodiversity-mediated benefits for crop production. Sci Adv. 5.

**Dainese, M., V. Riedinger, A. Holzschuh, D. Kleijn, J. Scheper, and I. Steffan-Dewenter**. **2018**. Managing trap-nesting bees as crop pollinators: Spatiotemporal effects of floral resources and antagonists. Journal of Applied Ecology. 55: 195–204.

**Doré, M., C. Fontaine, and E. Thébault**. **2021**. Relative effects of anthropogenic pressures, climate, and sampling design on the structure of pollination networks at the global scale. Glob Chang Biol. 27: 1266–1280.

**Feltham, H., K. Park, J. Minderman, and D. Goulson**. **2015**. Experimental evidence that wildflower strips increase pollinator visits to crops. Ecol Evol. 5: 3523–3530.

**Ferreira, P. A., D. Boscolo, and B. Felipe Viana**. **2013**. What do we know about the effects of landscape changes on plant-pollinator interaction networks? Ecol Indic. 31: 35–40.

**Fijen, T. P. M., J. A. Scheper, T. M. Boom, N. Janssen, I. Raemakers, and D. Kleijn**. **2018**. Insect pollination is at least as important for marketable crop yield as plant quality in a seed crop. Ecol Lett. 21: 1704–1713.

**Filipiak, M.** **2019**. Key pollen host plants provide balanced diets for wild bee larvae: A lesson for planting flower strips and hedgerows. J Appl Ecol. 56: 1410–1418.

**Fründ, J., K. E. Linsenmair, and N. Blüthgen**. **2010**. Pollinator diversity and specialization in relation to flower diversity. Oikos. 119: 1581–1590.

**Gardner, E., T. D. Breeze, Y. Clough, H. G. Smith, K. C. R Baldock, A. Campbell, M. P. D Garratt, M. A. K Gillespie, W. E. Kunin, M. McKerchar, S. G. Potts, D. Senapathi, G. N. Stone, F. Wäckers, D. B. Westbury, A. Wilby, and T. H. Oliver**. **2021**. Field boundary features can stabilise bee populations and the pollination of mass-flowering crops in rotational systems. J Appl Ecol. 58: 2287–2304.

**Garibaldi, L. A., L. G. Carvalheiro, S. D. Leonhardt, M. A. Aizen, B. R. Blaauw, R. Isaacs, M. Kuhlmann, D. Kleijn, A. M. Klein, C. Kremen, L. Morandin, J. Scheper, and R. Winfree**. **2014**. From research to action: enhancing crop yield through wild pollinators. Ecology and the Environment. 12: 439–447.

**Garibaldi, L. A., L. G. Carvalheiro, B. E. Vaissière, B. Gemmill-Herren, J. Hipólito, B. M. Freitas, H. T. Ngo, N. Azzu, A. Sáez, J. Åström, J. An, B. Blochtein, D. Buchori, F. J. Chamorro García, F. O. Da Silva, K. Devkota, M. De Fátima Ribeiro, L. Freitas, M. C. Gaglianone, M. Goss, M. Irshad, M. Kasina, A. J. S. Pacheco Filho, L. H. Piedade Kiill, P. Kwapong, G. N. Parra, C. Pires, V. Pires, R. S. Rawal, A. Rizali, A. M. Saraiva, R. Veldtman, B. F. Viana, S. Witter, and H. Zhang**. **2016**. Mutually beneficial pollinator diversity and crop yield outcomes in small and large farms. Science (1979). 351: 388–391.

**Garibaldi, L. A., I. Steffan-Dewenter, C. Kremen, J. M. Morales, R. Bommarco, S. A. Cunningham, L. G. Carvalheiro, N. P. Chacoff, J. H. Dudenhöffer, S. S. Greenleaf, A. Holzschuh, R. Isaacs, K. Krewenka, Y. Mandelik, M. M. Mayfield, L. A. Morandin, S. G. Potts, T. H. Ricketts, H. Szentgyörgyi, B. F. Viana, C. Westphal, R. Winfree, and A. M. Klein**. **2011**. Stability of pollination services decreases with isolation from natural areas despite honey bee visits. Ecol Lett. 14: 1062–1072.

**Garratt, M. P. D., R. Brown, C. Hartfield, A. Hart, S. G. Potts, and M. P. D. Garratt**. **2018**. Integrated crop pollination to buffer spatial and temporal variability in pollinator activity. Basic Appl Ecol. 32: 77–85.

**Garratt, M. P. D., D. Senapathi, D. J. Coston, S. R. Mortimer, and S. G. Potts**. **2017**. The benefits of hedgerows for pollinators and natural enemies depends on hedge quality and landscape context. Agric Ecosyst Environ. 247: 363–370.

**Gilpin, A. M., A. J. Denham, and D. J. Ayre**. **2019**. Do mass flowering agricultural species affect the pollination of Australian native plants through localised depletion of pollinators or pollinator spillover effects? Agric Ecosyst Environ. 277: 83–94.

**Grab, H., M. G. Branstetter, N. Amon, K. R. Urban-Mead, M. G. Park, J. Gibbs, E. J. Blitzer, K. Poveda, G. Loeb, and B. N. Danforth**. **2019**. Agriculturally dominated landscapes reduce bee phylogenetic diversity and pollination services. Science (1979). 363: 282–284.

**Grab, H., J. Brokaw, E. Anderson, L. Gedlinske, J. Gibbs, J. Wilson, G. Loeb, R. Isaacs, and K. Poveda**. **2019**. Habitat enhancements rescue bee body size from the negative effects of landscape simplification. Journal of Applied Ecology. 56: 2144–2154.

**Grab, H., J. Brokaw, E. Anderson, L. Gedlinske, J. Gibbs, J. Wilson, G. Loeb, | Rufus Isaacs, and K. Poveda**. **2019**. Habitat enhancements rescue bee body size from the negative effects of landscape simplification. J Appl Ecol. 56: 2144–2154.

**Grass, I., B. Jauker, I. Steffan-Dewenter, T. Tscharntke, and F. Jauker**. **2018**. Past and potential future effects of habitat fragmentation on structure and stability of plant–pollinator and host–parasitoid networks. Nat Ecol Evol. 2: 1408–1417.

**Greenleaf, S. S., and C. Kremen**. **2006**. Wild bee species increase tomato production and respond differently to surrounding land use in Northern California. Biol Conserv. 133: 81–87.

**Griffiths-Lee, J., E. Nicholls, and D. Goulson**. **2020**. Companion planting to attract pollinators increases the yield and quality of strawberry fruit in gardens and allotments. Ecol Entomol. 45: 1025–1034.

**Hardman, C. J., K. Norris, T. D. Nevard, B. Hughes, and S. G. Potts**. **2016**. Delivery of floral resources and pollination services on farmland under three different wildlife-friendly schemes. Agric Ecosyst Environ. 220: 142–151.

**Heller, S., N. K. Joshi, T. Leslie, E. G. Rajotte, and D. J. Biddinger**. **2019**. Diversified floral resource plantings support bee communities after apple bloom in commercial orchards. Sci Rep. 9: 17232.

**Holzschuh, A., J.-H. Dudenhöffer, and T. Tscharntke**. **2012**. Landscapes with wild bee habitats enhance pollination, fruit set and yield of sweet cherry. Biol Conserv. 153: 101–107.

**Horth, L., and L. A. Campbell**. **2018**. Supplementing small farms with native mason bees increases strawberry size and growth rate. Journal of Applied Ecology. 55: 591–599.

**Huang, H., and P. D’Odorico**. **2020**. Critical Transitions in Plant-Pollinator Systems Induced by Positive Inbreeding-Reward-Pollinator Feedbacks. iScience. 23.

**Indah Setyawati, A., R. Wijayanti, and R. Bandriyati Arni Putri**. **2014**. The role of flowering plants, Hibiscus sabdariffa and Crotalaria juncea in coffee ecosystem to diversity of insect pollinators and coffee fruit set. AIP Conf Proc. 20027.

**Jönsson, A. M., J. Ekroos, J. Dänhardt, G. K. S. Andersson, O. Olsson, and H. G. Smith**. **2015**. Sown flower strips in southern Sweden increase abundances of wild bees and hoverflies in the wider landscape. Biol Conserv. 184: 51–58.

**Kay, S., E. Kühn, M. Albrecht, L. Sutter, E. Szerencsits, and F. Herzog**. **2020**. Agroforestry can enhance foraging and nesting resources for pollinators with focus on solitary bees at the landscape scale. Agroforestry Systems. 94: 379–387.

**Kennedy, C. M., E. Lonsdorf, M. C. Neel, N. M. Williams, T. H. Ricketts, R. Winfree, R. Bommarco, C. Brittain, A. L. Burley, D. Cariveau, L. G. ısa Carvalheiro, N. P. Chacoff, S. A. Cunningham, B. N. Danforth, J.-H. Dudenh, E. Elle, H. R. Gaines, L. A. Garibaldi, C. Gratton, A. Holzschuh, R. Isaacs, S. K. Javorek, S. Jha, A. M. Klein, K. Krewenka, Y. Mandelik, M. M. Mayfield, L. Morandin, L. A. Neame, M. Otieno, M. Park, S. G. Potts, M. Rundl, A. Saez, I. Steffan-Dewenter, H. Taki, B. Felipe Viana, C. Westphal, J. K. Wilson, S. S. Greenleaf, and C. Kremen**. **2013**. A global quantitative synthesis of local and landscape effects on wild bee pollinators in agroecosystems. Ecol Lett. 16: 584–599.

**Kline, O., and N. K. Joshi**. **2020**. Mitigating the effects of habitat loss on solitary bees in agricultural ecosystems. Agriculture (Switzerland).

**Knapp, J. L., R. F. Shaw, and J. L. Osborne**. **2019**. Pollinator visitation to mass-flowering courgette and co-flowering wild flowers: Implications for pollination and bee conservation on farms. Basic Appl Ecol. 34: 85–94.

**Kordbacheh, F., M. Liebman, and M. Harris**. **2020**. Strips of prairie vegetation placed within row crops can sustain native bee communities. PLoS One. 15.

**Korpela, E. L., T. Hyvönen, S. Lindgren, and M. Kuussaari**. **2013**. Can pollination services, species diversity and conservation be simultaneously promoted by sown wildflower strips on farmland? Agric Ecosyst Environ. 179: 18–24.

**Lázaro, A., and D. Alomar**. **2019**. Landscape heterogeneity increases the spatial stability of pollination services to almond trees through the stability of pollinator visits. Agric Ecosyst Environ. 279: 149–155.

**Lichtenberg, E. M., C. M. Kennedy, C. Kremen, P. Batáry, F. Berendse, R. Bommarco, N. A. Bosque-Pérez, L. G. Carvalheiro, W. E. Snyder, N. M. Williams, R. Winfree, B. K. Klatt, S. Åström, F. Benjamin, C. Brittain, R. Chaplin-Kramer, Y. Clough, B. Danforth, T. Diekötter, S. D. Eigenbrode, J. Ekroos, E. Elle, B. M. Freitas, Y. Fukuda, H. R. Gaines-Day, H. Grab, C. Gratton, A. Holzschuh, R. Isaacs, M. Isaia, S. Jha, D. Jonason, V. P. Jones, A. M. Klein, J. Krauss, D. K. Letourneau, S. Macfadyen, R. E. Mallinger, E. A. Martin, E. Martinez, J. Memmott, L. Morandin, L. Neame, M. Otieno, M. G. Park, L. Pfiffner, M. J. O. Pocock, C. Ponce, S. G. Potts, K. Poveda, M. Ramos, J. A. Rosenheim, M. Rundlöf, H. Sardiñas, M. E. Saunders, N. L. Schon, A. R. Sciligo, C. S. Sidhu, I. Steffan-Dewenter, T. Tscharntke, M. Veselý, W. W. Weisser, J. K. Wilson, and D. W. Crowder**. **2017**. A global synthesis of the effects of diversified farming systems on arthropod diversity within fields and across agricultural landscapes. Glob Chang Biol. 23: 4946–4957.

**Liu, R., D. Chen, S. Luo, S. Xu, H. Xu, X. Shi, and Y. Zou**. **2020**. Quantifying pollination efficiency of flower-visiting insects and its application in estimating pollination services for common buckwheat. Agric Ecosyst Environ. 301.

**Mach, B. M., and D. A. Potter**. **2018**. Quantifying bee assemblages and attractiveness of flowering woody landscape plants for urban pollinator conservation. PLoS One. 13: e0208428.

**Main, A. R., E. B. Webb, K. W. Goyne, and D. Mengel**. **2020**. Reduced species richness of native bees in field margins associated with neonicotinoid concentrations in non-target soils. Agric Ecosyst Environ. 287.

**Marja, R., D. Kleijn, T. Tscharntke, A. M. Klein, T. Frank, and P. Batáry**. **2019**. Effectiveness of agri-environmental management on pollinators is moderated more by ecological contrast than by landscape structure or land-use intensity. Ecol Lett.

**Martin, E. A., M. Dainese, Y. Clough, A. Báldi, R. Bommarco, V. Gagic, M. P. D. Garratt, A. Holzschuh, D. Kleijn, A. Kovács-Hostyánszki, L. Marini, S. G. Potts, H. G. Smith, D. Al Hassan, M. Albrecht, G. K. S. Andersson, J. D. Asís, S. Aviron, M. V. Balzan, L. Baños-Picón, I. Bartomeus, P. Batáry, F. Burel, B. Caballero-López, E. D. Concepción, V. Coudrain, J. Dänhardt, M. Diaz, T. Diekötter, C. F. Dormann, R. Duflot, M. H. Entling, N. Farwig, C. Fischer, T. Frank, L. A. Garibaldi, J. Hermann, F. Herzog, D. Inclán, K. Jacot, F. Jauker, P. Jeanneret, M. Kaiser, J. Krauss, V. Le Féon, J. Marshall, A. C. Moonen, G. Moreno, V. Riedinger, M. Rundlöf, A. Rusch, J. Scheper, G. Schneider, C. Schüepp, S. Stutz, L. Sutter, G. Tamburini, C. Thies, J. Tormos, T. Tscharntke, M. Tschumi, D. Uzman, C. Wagner, M. Zubair-Anjum, and I. Steffan-Dewenter**. **2019**. The interplay of landscape composition and configuration: new pathways to manage functional biodiversity and agroecosystem services across Europe. Ecol Lett. 22: 1083–1094.

**McCallum, R. S., N. L. McLean, and G. C. Cutler**. **2021**. The impact of planting buckwheat strips along lowbush blueberry fields on beneficial insects. Canadian Journal of Plant Science. 101: 166–176.

**Medeiros, H. R., F. Martello, E. A. B. Almeida, X. Mengual, K. A. Harper, Y. C. Grandinete, J. P. Metzger, C. A. Righi, and M. C. Ribeiro**. **2019**. Landscape structure shapes the diversity of beneficial insects in coffee producing landscapes. Biol Conserv. 238: 108193.

**Mkenda, P. A., P. A. Ndakidemi, E. Mbega, P. C. Stevenson, S. E. J. Arnold, G. M. Gurr, and S. R. Belmain**. **2019**. Multiple ecosystem services from field margin vegetation for ecological sustainability in agriculture: Scientific evidence and knowledge gaps. PeerJ. 2019: 1–33.

**Morandin, L. A., and C. Kremen**. **2013**. Hedgerow restoration promotes pollinator populations and exports native bees to adjacent fields. Ecological Applications. 23: 829–839.

**Nichols, R. N., D. Goulson, and J. M. Holland**. **2019**. The best wildflowers for wild bees. J Insect Conserv. 23: 819–830.

**Nicholson, C. C., and T. H. Ricketts**. **2019**. Wild pollinators improve production, uniformity, and timing of blueberry crops. Agric Ecosyst Environ. 272: 29–37.

**Nicholson, C. C., T. H. Ricketts, I. Koh, H. G. Smith, E. V Lonsdorf, and O. Olsson**. **2019**. Flowering resources distract pollinators from crops: Model predictions from landscape simulations. J Appl Ecol. 56: 618–628.

**Ouvrard, P., and A. L. Jacquemart**. **2018**. Agri-environment schemes targeting farmland bird populations also provide food for pollinating insects. Agric For Entomol. 20: 558–574.

**Papanikolaou, A. D., I. Kuhn, M. Frenzel, M. Kuhlmann, P. Poschlod, S. G. Potts, S. P. M. Roberts, and O. Schweiger**. **2017**. Wild bee and floral diversity co-vary in response to the direct and indirect impacts of land use. Ecosphere. 8.

**Papanikolaou, A. D., I. Kühn, M. Frenzel, and O. Schweiger**. **2017**. Semi-natural habitats mitigate the effects of temperature rise on wild bees. Journal of Applied Ecology. 54: 527–536.

**Paterson, C., K. Cottenie, and A. S. Macdougall**. **2019**. Restored native prairie supports abundant and species-rich native bee communities on conventional farms. Restor Ecol. 27: 1291–1299.

**Peters, V. E., and C. R. Carroll**. **2012**. Temporal variation in coffee flowering may influence the effects of bee species richness and abundance on coffee production. Agroforestry Systems. 85: 95–103.

**Porto, R. G., R. F. de Almeida, O. Cruz-Neto, M. Tabarelli, B. F. Viana, C. A. Peres, and A. V. Lopes**. **2020**. Pollination ecosystem services: A comprehensive review of economic values, research funding and policy actions. Food Secur. 12: 1425–1442.

**Pywell, R. F., M. S. Heard, B. A. Woodcock, S. Hinsley, L. Ridding, M. Nowakowski, and J. M. Bullock**. **2015**. Wildlife-friendly farming increases crop yield: Evidence for ecological intensification. Proceedings of the Royal Society B: Biological Sciences. 282.

**Rader, R., S. A. Cunningham, B. G. Howlett, and D. W. Inouye**. **2020**. Non-bee insects as visitors and pollinators of crops: Biology, ecology, and management. Annu Rev Entomol. 65: 391–407.

**Redhead, J. W., B. A. Woodcock, M. J. O. Pocock, R. F. Pywell, A. J. Vanbergen, and T. H. Oliver**. **2018**. Potential landscape-scale pollinator networks across Great Britain: structure, stability and influence of agricultural land cover. Ecol Lett. 21: 1821–1832.

**Reilly, J. R., D. R. Artz, D. Biddinger, K. Bobiwash, N. K. Boyle, C. Brittain, J. Brokaw, J. W. Campbell, J. Daniels, E. Elle, J. D. Ellis, S. J. Fleischer, J. Gibbs, R. L. Gillespie, K. B. Gundersen, L. Gut, G. Hoffman, N. Joshi, O. Lundin, K. Mason, C. M. Mcgrady, S. S. Peterson, T. L. Pitts-Singer, S. Rao, N. Rothwell, L. Rowe, K. L. Ward, N. M. Williams, J. K. Wilson, R. Isaacs, and R. Winfree**. **2020**. Crop production in the USA is frequently limited by a lack of pollinators. Proceedings of the Royal Society B. 287.

**Reverté, S., J. Bosch, X. Arnan, T. Roslin, C. Stefanescu, J. A. Calleja, R. Molowny-Horas, C. Hernández-Castellano, and A. Rodrigo**. **2019**. Spatial variability in a plant-pollinator community across a continuous habitat: high heterogeneity in the face of apparent uniformity. Ecography. 42: 1558–1568.

**Rollin, O., N. Pérez-Méndez, V. Bretagnolle, and M. Henry**. **2019**. Preserving habitat quality at local and landscape scales increases wild bee diversity in intensive farming systems. Agric Ecosyst Environ. 275: 73–80.

**Rollings, R., and D. Goulson**. **2019**. Quantifying the attractiveness of garden flowers for pollinators. J Insect Conserv. 23: 803–817.

**Ropars, L., L. Affre, L. Schurr, F. Flacher, D. Genoud, C. Mutillod, and B. Geslin**. **2020**. Land cover composition, local plant community composition and honeybee colony density affect wild bee species assemblages in a Mediterranean biodiversity hot-spot. Acta Oecologica. 104: 103546.

**Rundlöf, M., O. Lundin, and R. Bommarco**. **2018**. Annual flower strips support pollinators and potentially enhance red clover seed yield. Ecol Evol. 8: 7974–7985.

**Saunders, M. E., and R. Rader**. **2019**. Network modularity influences plant reproduction in a mosaic tropical agroecosystem. Proc Biol Sci. 286: 20190296.

**Schwarz, B., D. P. Vázquez, P. J. CaraDonna, T. M. Knight, G. Benadi, C. F. Dormann, B. Gauzens, E. Motivans, J. Resasco, N. Blüthgen, L. A. Burkle, Q. Fang, C. N. Kaiser-Bunbury, R. Alarcón, J. A. Bain, N. P. Chacoff, S. Q. Huang, G. LeBuhn, M. MacLeod, T. Petanidou, C. Rasmussen, M. P. Simanonok, A. H. Thompson, and J. Fründ**. **2020**. Temporal scale-dependence of plant–pollinator networks. Oikos. 129: 1289–1302.

**Senapathi, D., L. G. Carvalheiro, J. C. Biesmeijer, C. A. Dodson, R. L. Evans, M. McKerchar, D. R. Morton, E. D. Moss, S. P. M. Roberts, W. E. Kunin, and S. G. Potts**. **2015**. The impact of over 80 years of land cover changes on bee and wasp pollinator communities in England. Proceedings of the Royal Society B: Biological Sciences. 282.

**Shaw, R. F., B. B. Phillips, T. Doyle, J. K. Pell, J. W. Redhead, J. Savage, B. A. Woodcock, J. M. Bullock, and J. L. Osborne**. **2020**. Mass-flowering crops have a greater impact than semi-natural habitat on crop pollinators and pollen deposition. Landsc Ecol. 35: 513–527.

**Shuler, R. E., H. Roulston, G. E. Farris, and T. ’ai**. **2005**. Farming Practices Influence Wild Pollinator Populations on Squash and Pumpkin. J. Econ. Entomol. 98: 790–795.

**Sritongchuay, T., A. C. Hughes, J. Memmott, and S. Bumrungsri**. **2019**. Forest proximity and lowland mosaic increase robustness of tropical pollination networks in mixed fruit orchards. Landsc Urban Plan. 192.

**Timberlake, T. P., I. P. Vaughan, and J. Memmott**. **2019**. Phenology of farmland floral resources reveals seasonal gaps in nectar availability for bumblebees. Journal of Applied Ecology. 56: 1585–1596.

**Tonietto, R. K., and D. J. Larkin**. **2018**. Habitat restoration benefits wild bees: A meta-analysis. Journal of Applied Ecology. 55: 582–590.

**Uyttenbroeck, R., J. Piqueray, S. Hatt, G. Mahy, and A. Monty**. **2017**. Increasing plant functional diversity is not the key for supporting pollinators in wildflower strips. Agric Ecosyst Environ. 249: 144–155.

**Uzman, D., A. Reineke, M. H. Entling, and I. Leyer**. **2020**. Habitat area and connectivity support cavity-nesting bees in vineyards more than organic management. Biol Conserv. 242.

**Vanderplanck, M., B. Martinet, L. G. Carvalheiro, P. Rasmont, A. Barraud, C. Renaudeau, and D. Michez**. **2019**. Ensuring access to high-quality resources reduces the impacts of heat stress on bees. Sci Rep. 9.

**Varah, A., H. Jones, J. Smith, and S. G. Potts**. **2020**. Temperate agroforestry systems provide greater pollination service than monoculture. Agric Ecosyst Environ. 301.

**Venturini, E. M., F. A. Drummond, A. K. Hoshide, A. C. Dibble, and L. B. Stack**. **2017**. Pollination reservoirs for wild bee habitat enhancement in cropping systems: a review. Agroecology and Sustainable Food Systems. 41: 101–142.

**Wietzke, A., C. Westphal, P. Gras, M. Kraft, K. Pfohl, P. Karlovsky, E. Pawelzik, T. Tscharntke, and I. Smit**. **2018**. Insect pollination as a key factor for strawberry physiology and marketable fruit quality. Agric Ecosyst Environ. 258: 197–204.

**Winfree, R., N. M. Williams, H. Gaines, J. S. Ascher, and C. Kremen**. **2008**. Wild bee pollinators provide the majority of crop visitation across land-use gradients in New Jersey and Pennsylvania, USA. Journal of Applied Ecology. 45: 793–802.

**Wood, T. J., J. M. Holland, and D. Goulson**. **2015**. Pollinator-friendly management does not increase the diversity of farmland bees and wasps. Biol Conserv. 187: 120–126.

**Wood, T. J., J. M. Holland, and D. Goulson**. **2017**. Providing foraging resources for solitary bees on farmland: current schemes for pollinators benefit a limited suite of species. Journal of Applied Ecology. 54: 323–333.

**Woodcock, B. A., M. P. D. Garratt, G. D. Powney, R. F. Shaw, J. L. Osborne, J. Soroka, S. A. M. Lindström, D. Stanley, P. Ouvrard, M. E. Edwards, F. Jauker, M. E. McCracken, Y. Zou, S. G. Potts, M. Rundlöf, J. A. Noriega, A. Greenop, H. G. Smith, R. Bommarco, W. van der Werf, J. C. Stout, I. Steffan-Dewenter, L. Morandin, J. M. Bullock, and R. F. Pywell**. **2019**. Meta-analysis reveals that pollinator functional diversity and abundance enhance crop pollination and yield. Nat Commun. 10.

**Zamorano, J., I. Bartomeus, A. A. Grez, and L. A. Garibaldi**. **2020**. Field margin floral enhancements increase pollinator diversity at the field edge but show no consistent spillover into the crop field: a meta-analysis. Insect Conserv Divers. 13: 519–531.
